# Chronic Morphine Administration Differentially Modulates Viral Reservoirs in a Simian Immunodeficiency Virus SIVmac251-Infected Rhesus Macaque Model

**DOI:** 10.1128/JVI.01657-20

**Published:** 2021-02-10

**Authors:** Arpan Acharya, Omalla A. Olwenyi, Michellie Thurman, Kabita Pandey, Brenda M. Morsey, Benjamin Lamberty, Natasha Ferguson, Shannon Callen, Qiu Fang, Shilpa J. Buch, Howard S. Fox, Siddappa N. Byrareddy

**Affiliations:** a Department of Pharmacology and Experimental Neuroscience, University of Nebraska Medical Center, Omaha, Nebraska, USA; b Department of Neurological Sciences, University of Nebraska Medical Center, Omaha, Nebraska, USA; c Department of Biostatistics, College of Public Health, University of Nebraska Medical Center, Omaha, Nebraska, USA; Emory University

**Keywords:** opioid use disorder (OUD), combined antiretroviral therapy (cART), SIV reservoirs, Tat/Rev induced limited dilution assay (TILDA), quantitative viral outgrowth assay (QVOA), intact proviral DNA assay (IPDA)

## Abstract

Human immunodeficiency virus (HIV) persists in cellular reservoirs despite effective combined antiretroviral therapy (cART), and there is viremia flare-up upon therapy interruption. Opioids modulate the immune system and suppress antiviral gene responses, which significantly impact people living with HIV (PLWH). However, the effect of opioids on viral reservoir dynamics remains elusive. Here, we developed a morphine-dependent simian immunodeficiency virus SIVmac251-infected rhesus macaque (RM) model to study the impact of opioids on HIV reservoirs. RMs on a morphine (or saline control) regimen were infected with SIVmac251. cART was initiated in approximately half the animals 5 weeks postinfection, and morphine/saline administration continued until the end of the study. Among the untreated RMs, we did not find any difference in plasma/cerebrospinal fluid (CSF) viral loads or in cell-associated DNA/RNA viral loads in anatomical tissues. On the other hand, within the cART-suppressed macaques, there was a reduction in cell-associated DNA load, intact proviral DNA levels, and in inducible SIV reservoirs in lymph nodes (LNs) of morphine-administered RMs. In distinction to those in LNs, in the central nervous system (CNS), the size of latent SIV reservoirs was larger in the CD11b^+^ microglia/macrophages in morphine-dependent RMs. These results suggest that in the proposed model, morphine plays a differential role in SIV reservoirs by reducing the CD4^+^ T-cell reservoir in lymphoid tissues while increasing the microglia/macrophage reservoir size in CNS tissue. The findings from this preclinical model will serve as a tool for screening therapeutic strategies to reduce/eliminate HIV reservoirs in opioid-dependent PLWH.

**IMPORTANCE** Identification and clearance of human immunodeficiency virus (HIV) reservoirs is a major challenge in achieving a cure for HIV. This is further complicated by comorbidities that may alter the size of the reservoirs. There is an overlap between the risk factors for HIV and opioid abuse. Opiate abuse has been recognized as a prominent comorbidity in HIV-infected populations. People infected with HIV also abusing opioids have immune modulatory effects and more severe neurological disease. However, the impact of opioid abuse on HIV reservoirs remains unclear. In this study, we used a morphine-dependent simian immunodeficiency virus SIVmac251-infected rhesus macaque (RM) model to study the impact of opioids on HIV reservoirs. Our studies suggested that people with HIV who abuse opioids have higher reservoirs in central nervous system (CNS) than in the lymphoid system. Extrapolating the macaque findings to humans suggests that such differential modulation of HIV reservoirs among people living with HIV abusing opioids could be considered for future HIV cure research efforts.

## Abstract (precis)

Identification and clearance of human immunodeficiency virus (HIV) reservoirs is a major challenge in achieving a cure for HIV. This is further complicated by comorbidities that may alter the size of the reservoirs.

## INTRODUCTION

Opioid use disorder (OUD) is a chronic illness characterized by persistent use of opioids to the detriment of the user (1). Globally, there was an estimated 26.8 million people living with OUD in 2016 (1), of which 2.1 million were from the United States (2). People living with OUD are at higher risk of medical comorbidities, including human immunodeficiency virus (HIV) and hepatitis C virus (HCV) infection (3). Worldwide, it is estimated that 80% of injection drug users take opioids, and 17.8% of them are HIV positive (4, 5). Epidemiological studies have shown that chronic opioid administration is immunosuppressive, downregulates the antiviral genes, and increases the susceptibility to infections such as tuberculosis and HIV (6–12). The innate and adaptive immune responses act in synchrony against the invading pathogens, and opioids acting through the opioid receptors alter both these responses (13, 14).

OUD is important in the context of HIV infection, as several studies have reported that people living with HIV (PLWH) who use opioids are at a higher risk of developing neurological disorders than PLWH without OUD (15–20). Opioids have been shown to upregulate the expression of CCR5 while downregulating the expression of its cognate β-chemokine production by acting through µ-opioid receptors in macrophages and microglia, thereby boosting the entry of R5 tropic viruses in these target cells (21–24). Opioids and HIV synergistically act to activate glia and upregulate the expression of cytokines and chemokines, leading, in turn, to neuronal damage and development of hyperalgesia (25–27).

One barrier to the cure of HIV is that the HIV genome integrates into the host chromosome (28). A subset of infected cells with integrated proviral DNA can enter a transcriptionally silent state that can persist in the context of combination antiretroviral therapy (cART) and escape host cell immune surveillance owing to its inability to produce antigens essential for triggering immune responses. This, in turn, leads to creation of a pool of long-lived viral reservoirs (29). HIV reservoirs are seeded early after infection and persist through the entire lifetime of the host. Among all the cellular reservoirs, resting memory CD4^+^ T cells are the best characterized, though persistence of viral reservoirs in perivascular macrophages and microglia in the central nervous system (CNS) has also been reported (30, 31). Active and latent reservoirs of HIV also persist in several other tissues owing to the presence of subtherapeutic concentrations of antiretrovirals in these tissue locations. The major barriers to penetration of ART in deep tissues are their physiochemical properties and the presence of drug efflux transporters (32) among the well-characterized tissue reservoirs such as lymph nodes (LNs), spleen, gut-associated lymphoid tissues (GALT), and the thymus (33). The other less-characterized anatomical tissue reservoirs of HIV include the kidney, liver, lung, bone marrow, genital tract, and CNS (34–36).

Morphine and other frequently used/abused opioids have been shown to inhibit the phagocytic property of macrophages and the migration of neutrophils and have a cytopathic effect on NK cells, which, in combination, results in dampening of the innate immune system (37). During adaptive immune responses, chronic morphine exposure leads to defective antigen presentation and a phenotype switch from Th1 to Th2 in CD4^+^ T cells, resulting in inhibition of proinflammatory responses that are critical for eradicating invading intracellular pathogens (38). Interestingly, there are also conflicting reports refuting the immune suppressive effects of morphine, with some reports from rodent models and studies using *ex vivo* human samples indicating activation of the pattern recognition receptor Toll-like receptor 4 (TLR4) and subsequent cellular activation (39–43).

There are multiple lines of evidence implicating the role of morphine in mediating immunosuppressive effects in the periphery, its roles in infectivity, transmission, and pathogenesis of HIV, and its potentiation of HIV neuropathogenesis (16, 21, 24, 44, 45). However, the role of morphine in modulating the persistence of HIV reservoirs in various anatomical sanctuaries remains elusive. In the present study, we sought to examine the impact of morphine dependency on the size of simian immunodeficiency virus (SIV) reservoirs using the SIVmac251-infected rhesus macaque (RM) model. SIV reservoirs were found to persist in all the major cellular and tissue locations in ART-suppressed, morphine-dependent, and saline-administered rhesus macaques. Our findings suggest that morphine differentially modulates the size of viral reservoirs in lymphoid tissues versus those in the CNS. To the best of our knowledge, this is the first study to report differential regulation of viral reservoir dynamics in the context of opioid dependence and warrants further investigation to dissect the molecular mechanism(s).

## RESULTS

### Dynamics of plasma and cerebrospinal fluid viral loads in morphine-dependent versus control SIVmac251-infected rhesus macaques with and without antiretroviral therapy.

**FIG 1:**
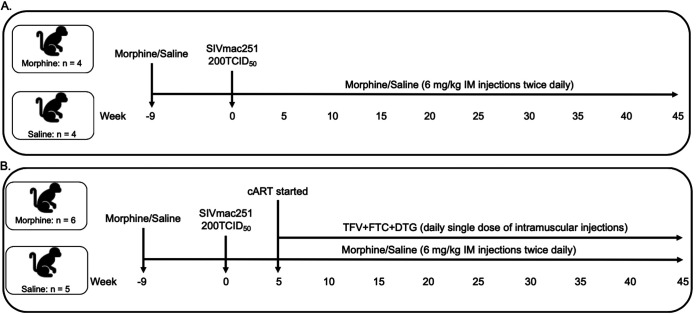
Experimental schema utilized for the study. The study included a total of 19 rhesus macaques. The study had two experimental arms, one of which contained eight monkeys that did not receive antiretroviral therapy (A), and the other group had 11 macaques that were treated with combinational antiretroviral therapy (B). (A) In this group of eight animals, four were ramped up with 6-mg/kg intramuscular (i.m.) injections of morphine twice daily for 2 weeks and then continued with morphine doses for 7 weeks, and the other four animals received similar doses of normal saline (control group). After 9 weeks, all the macaques were intravenously infected with 200 TCID_50_ of SIVmac251, while the administration of morphine/saline continued until end of the study. (B) In this group of 11 animals, six were ramp up with 6-mg/kg intramuscular injections of morphine twice daily for 2 weeks and then continued with morphine doses for 7 weeks, and the other five animals received similar doses of normal saline (control group). After 9 weeks, all the macaques were intravenously infected with 200 TCID_50_ of SIVmac251. Five weeks postinfection, antiretroviral therapy (ART) was initiated and continued until end of the study. ART regimen consisted of two reverse transcriptase inhibitors (FTC, 40 mg/ml, and TFV, 20 mg/ml) and one integrase inhibitor (DTG, 2.5 mg/ml). The antiretroviral drugs were administered subcutaneously once daily at 1 ml/kg body weight, while the administration of morphine/saline continued until end of the study.

**FIG 2:**
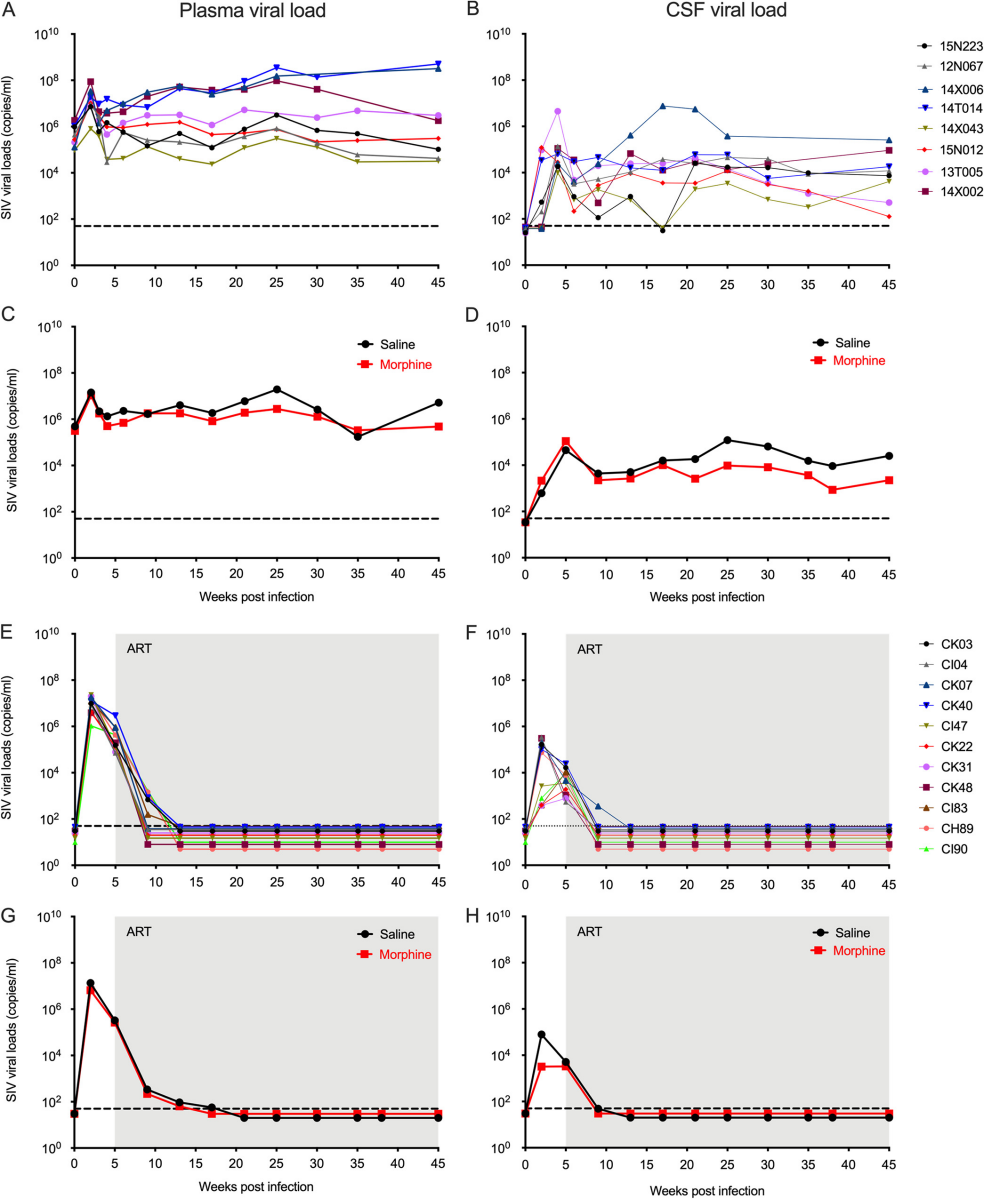
Plasma and CSF viral loads of morphine-administered SIVmac251-infected rhesus macaques. Ten macaques were ramped-up with twice daily doses of morphine (6 mg/kg) for 8 weeks, and nine macaques served as controls (received saline); the macaques were then infected with SIVmac251. Five weeks postinfection, in six animals from the morphine group and five animals from the control group, daily single doses of combination antiretroviral therapy (cART) were initiated. Daily doses of cART and morphine/saline administration continued until the end of the study. Plasma and CSF SIV viral loads were measured longitudinally in all rhesus macaques by using a quantitative PCR (qPCR) assay. (A) Longitudinal plasma viral loads in individual animals that did not receive cART (copies/ml). (B) Longitudinal CSF viral loads in individual animals that did not receive cART (copies/ml). (C) Geometric means of plasma viral loads in animals that did not receive cART (black line represents control group who received saline and red line represents morphine-treated group). (D) Geometric means of CSF viral loads in animals that did not receive cART (black line represents control group who received saline and red line represents morphine-treated group). (E) Longitudinal plasma viral loads in individual animals that received cART (copies/ml). (F) Longitudinal CSF viral loads in individual animals that received cART (copies/ml). (G) Geometric means of plasma viral loads in animals that received cART (black line represents control group who received saline and red line represents morphine-treated group). (H) Geometric means of CSF viral loads in animals that received cART (black line represents control group who received saline and red line represents morphine treated group). The shaded regions indicate ART phase of the study. The dashed lines represent the limit of detection of the assay (50 copies/ml).

In this study, we included a total of 19 macaques. Ten RMs were ramped up over 2 weeks to a final dosage of 6 mg/kg body weight morphine administered twice daily, which was maintained for 7 weeks, while nine RMs received saline and served as the control. At week 9, all the macaques were inoculated with a single 200 50% tissue culture infective dose (TCID_50_) of SIVmac251 by intravenous injection. Five weeks postinoculation, cART was initiated in six of ten RMs in the morphine group and in five of nine RMs in the control group. Four monkeys from each group were left untreated until the end of the study (Fig. 1). Within the control group (*n* = 9), the median peak plasma viral load was 1.25 × 10^7^ SIV copies/ml and median peak cerebrospinal fluid (CSF) viral load was 1.02 × 10^5^ SIV copies/ml. On the other hand, in the morphine-administered group (*n* = 10), the median peak plasma and CSF viral loads were 8.68 × 10^7^ SIV copies/ml and 4.62 × 10^4^ SIV copies/ml, respectively (Fig. 2A, B, E, and F). All 11 animals from the antiretroviral-treated group became aviremic with completely suppressed plasma and CSF viral loads within 13 weeks postinoculation, which is equivalent to 8 weeks of antiretroviral therapy, and they remained virally suppressed until the end of this study (Fig. 2E and F). The longitudinal geometric means of plasma and CSF viral loads between morphine and saline-treated RMs in all the groups are presented in Fig. 2C, D, G, and H.

### Cell-associated SIV DNA/RNA in the blood, lymph nodes, and rectal mucosal tissues.

**FIG 3:**
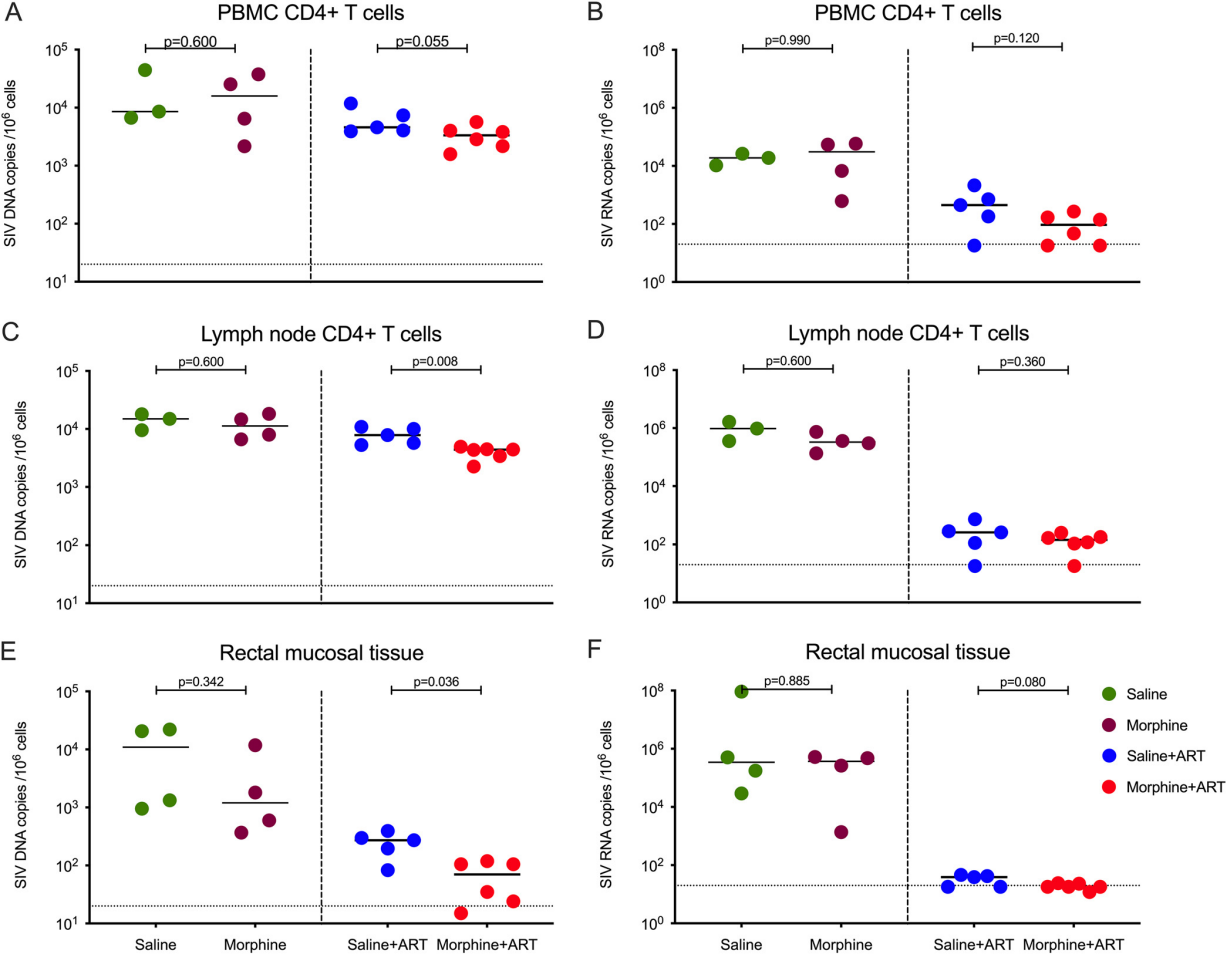
Cell-associated SIV DNA and RNA levels in different tissue compartments between morphine-administered and control groups of SIVmac251-infected rhesus macaques. (A) SIV DNA copies per million CD4^+^ T cells isolated from peripheral blood mononuclear cells. (B) SIV RNA copies per million CD4^+^ T cells isolated from peripheral blood mononuclear cells. (C) SIV DNA copies per million CD4^+^ T cells isolated from lymph node (LN) cells. (D) SIV RNA copies per million CD4^+^ T cells isolated from LN cells. (E) SIV DNA copies per million cells from rectal mucosal tissue. (F) SIV RNA copies per million cells from rectal mucosal tissue. The green dots represent samples from control animals administered saline that did not receive ART; maroon dots represent samples from morphine-administered animals that did not receive ART; blue dots represent samples from control animals administered saline and treated with ART; and the red dots represent samples from morphine-administered animals treated with ART. The horizontal dashed lines represent limit of detection of the assay (20 copies/ml). All values below the limit of detection were brought to the limit of detection for better representation in the figure. In the ART-naive control group, for one RM we were not able to recover enough cells from PBMC and lymph nodes for measuring cell-associated DNA/RNA.

To understand how chronic morphine administration could modulate the size of SIV reservoirs in various tissue compartments in the presence and/or absence of cART, we sought to quantify the cell-associated SIV DNA and RNA in various anatomical tissue sanctuaries collected during necropsy of all the RMs included in this study. Since CD4^+^ T cells are known to be the major contributor to viral reservoirs in both the blood and LNs, CD4^+^ T cells were purified from peripheral blood and LNs followed by isolation of DNA/RNA as described in Materials and Methods. In peripheral blood mononuclear cells (PBMCs) of ART-naive RMs, the median cell-associated SIV DNA load was 15,898 copies/10^6^ CD4^+^ T cells in the morphine-administered group versus 8,547 copies/10^6^ CD4^+^ T cells in saline controls (*P* = 0.600). In the PBMCs of ART-treated RMs, the median cell-associated SIV DNA load was 3,332 copies/10^6^ CD4^+^ T cells in the morphine-administered group versus 4,583 copies/10^6^ CD4^+^ T cells in saline controls (*P* = 0.055) (Fig. 3A). Similarly, in PBMCs of ART-naive RMs, the median cell-associated SIV RNA load was 30,533 copies/10^6^ CD4^+^ T cells in the morphine-administered group versus 18,890 copies/10^6^ CD4^+^ T cells in saline controls (*P* = 0.990). In the PBMCs of the ART-treated group, the median cell-associated SIV RNA load was 94 copies/10^6^ CD4^+^ T cells in the morphine-administered group versus 446 copies/10^6^ CD4^+^ T cells in controls (*P* = 0.120) (Fig. 3B).

Next, we measured the cell-associated DNA and RNA in CD4^+^ T cells purified from LNs. In LNs of ART-naive RMs, the median cell-associated SIV DNA load was 11,295 copies/10^6^ CD4^+^ T cells in the morphine-administered group versus 14,962 copies/10^6^ CD4^+^ T cells in saline controls (*P* = 0.600). On the other hand, in LNs of the ART group, a significant difference was found, with a median cell-associated SIV DNA load of 4,392 copies/10^6^ CD4^+^ T cells in the morphine-administered group versus 7,847 copies/10^6^ CD4^+^ T cells in saline controls (*P* = 0.008) (Fig. 3C). In LNs of ART-naive RMs, the median cell-associated SIV RNA load was 330,802 copies/10^6^ CD4^+^ T cells in the morphine-administered group versus 968,610 copies/10^6^ CD4^+^ T cells in saline controls (*P* = 0.220). In the LNs of the ART-treated group, the median cell-associated SIV RNA load was 143 copies/10^6^ CD4^+^ T cells in the morphine-administered group versus 260 copies/10^6^ CD4^+^ T cells in saline controls (*P* = 0.360) (Fig. 3D).

Furthermore, we assessed cell-associated DNA and RNA in mucosal tissue collected from the rectum during necropsy. In the ART-naive RMs, the median cell-associated SIV DNA load was 1,201 copies/10^6^ cells in the morphine-administered group versus 10,961 copies/10^6^ cells in saline controls (*P* = 0.342). In the ART-treated group, a significant difference was found, with a median cell-associated SIV DNA load of 70 copies/10^6^ cells in the morphine-administered group versus 272 copies/10^6^ cells in saline controls (*P* = 0.036) (Fig. 3E). In ART-naive RMs, the median cell-associated SIV RNA load was 368,990 copies/10^6^ cells in the morphine-administered group versus 341,883 copies/10^6^ cells in saline controls (*P* = 0.885). In the ART-treated group, the median cell-associated SIV RNA load was 11 copies/10^6^ cells in the morphine-administered group versus 39 copies/10^6^ cells in saline controls (*P* = 0.080) (Fig. 3F).

**FIG 4:**
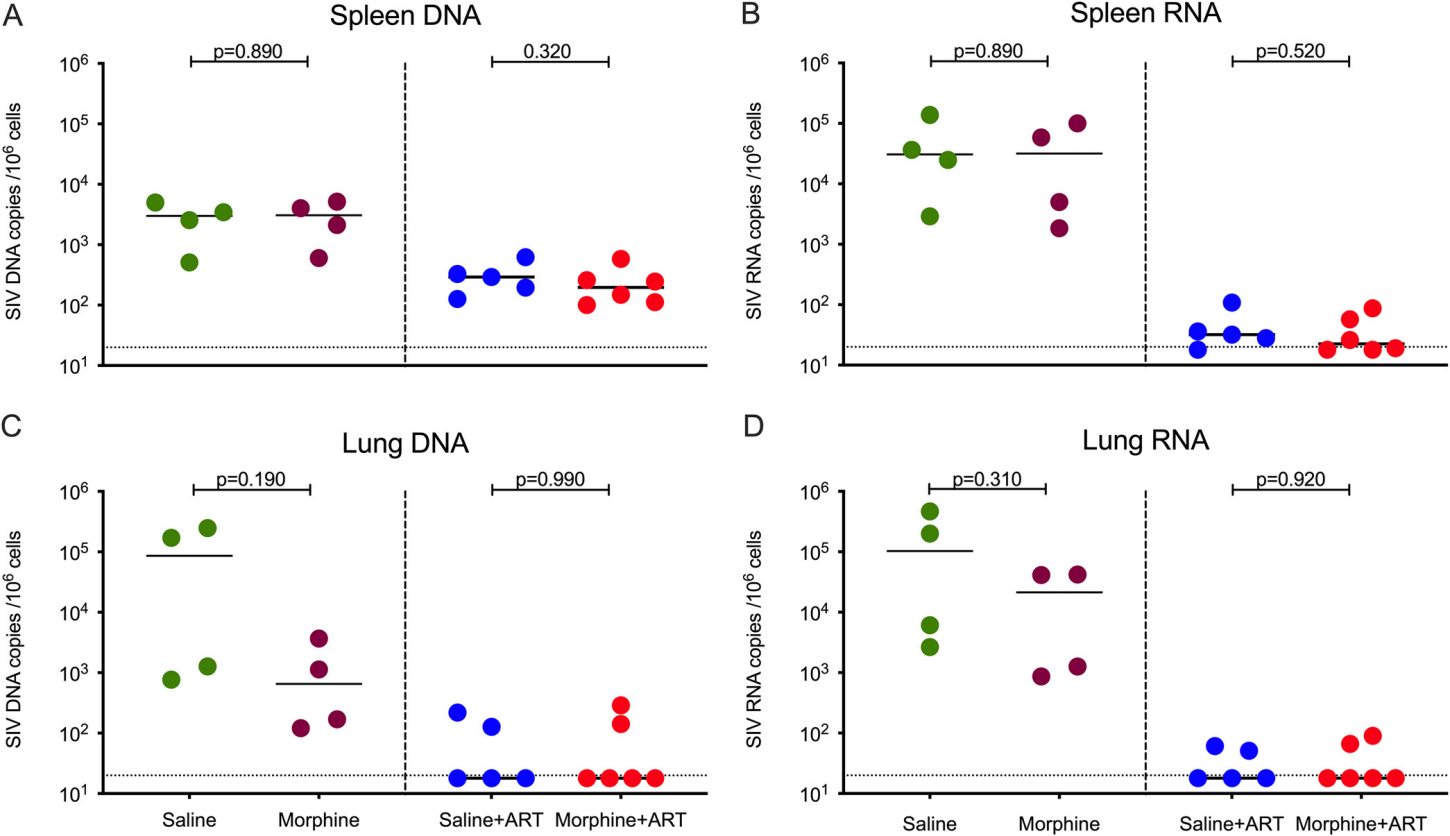
Cell-associated SIV DNA and RNA levels in spleen and lung in morphine-administered versus control groups of SIVmac251-infected rhesus macaques. (A) SIV DNA copies per million cells from spleen. (B) SIV RNA copies per million cells from spleen. (C) SIV DNA copies per million alveolar macrophages from lung. (D) SIV RNA copies per million alveolar macrophages from lung. The green dots represent samples from control animals administered saline that did not receive ART; maroon dots represent samples from morphine-administered animals that did not receive ART; blue dots represent samples from control animals administered saline and treated with ART; and the red dots represent samples from morphine-administered animals treated with ART. The horizontal dashed lines represent limit of detection of the assay (20 copies/ml). All values bellow the limit of detection were brought to the limit of detection for better representation in the figure.

We also measured the cell-associated DNA/RNA in both the spleens and lungs of macaques. There was no difference in cell-associated SIV DNA/RNA levels in the lungs or spleens of the morphine-administered versus saline controls in the presence of ART treatment as well as in the ART-naive RMs (Fig. 4). In summary, we found significant reduction in cell-associated DNA viral loads within LNs and rectal mucosal tissue in morphine-administered cART-treated animals compared with that in saline controls.

### Quantitation of SIV reservoirs in CD4^+^ T cells of blood and lymph nodes.

**FIG 5:**
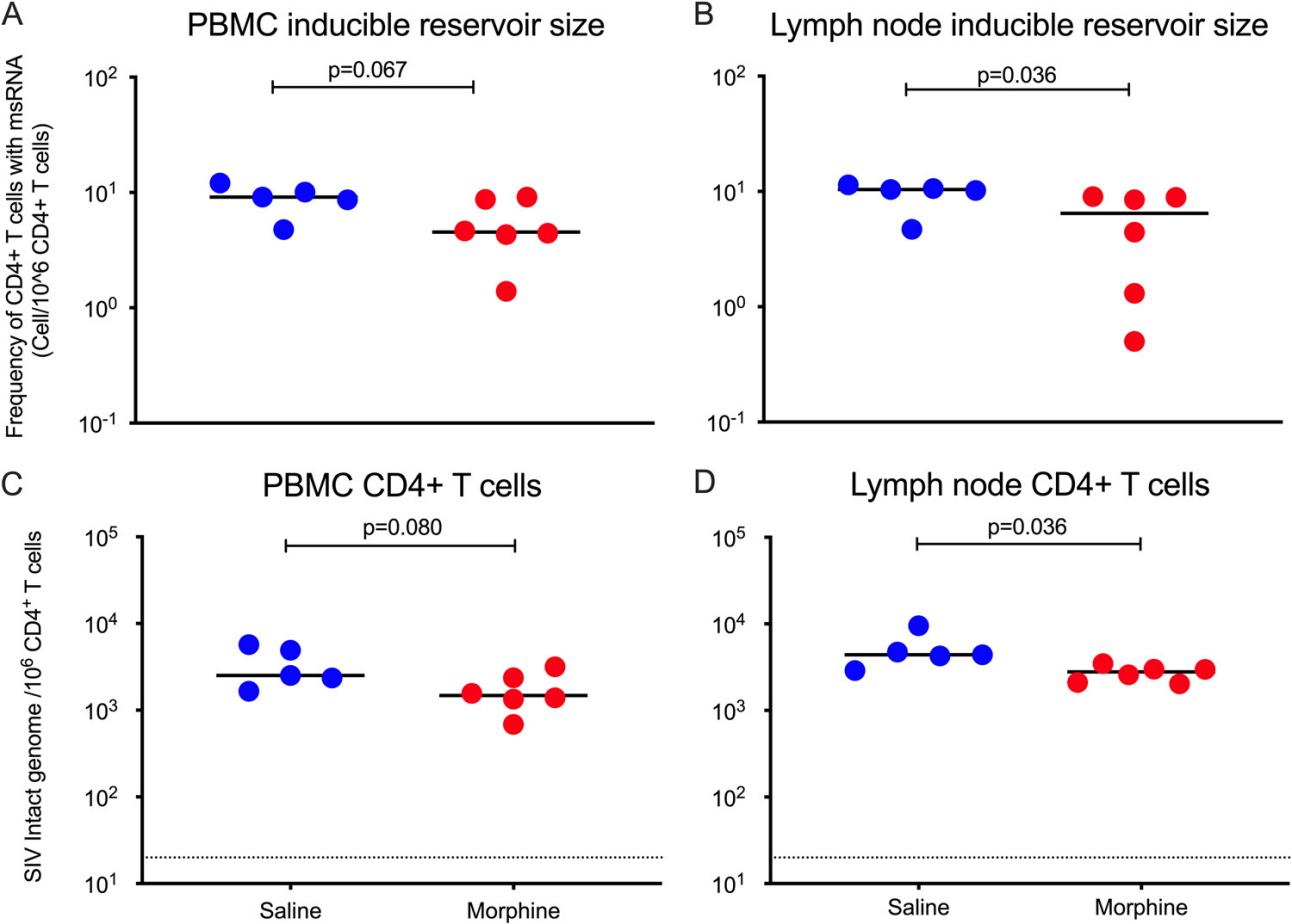
Size of SIV reservoirs in CD4^+^ T cells from peripheral blood and lymph nodes (LNs) in morphine-administered versus control group of SIVmac251-infected ART-suppressed rhesus macaques. (A and B) Sizes of inducible SIV latent reservoirs in CD4^+^ T cells from peripheral blood and LNs of morphine-administered and control rhesus macaques that received saline were measured by Tat/rev induced limiting dilution assay (TILDA), which quantifies the frequency of CD4^+^ T cells producing inducible ms-Tat/rev transcript per million of CD4^+^ T cells. (C and D) Frequencies of intact SIV genomes in CD4^+^ T cells isolated from blood and LNs of morphine/saline-administered, SIVmac251-infected cART-treated rhesus macaques were quantified using IPDA.

We used the Tat/rev induced limiting dilution assay (TILDA) to measure the size of inducible SIV reservoirs in the blood and LNs of morphine/saline-administered, SIV-infected cART-treated RMs (Fig. 1B). In peripheral blood, the median frequency of CD4^+^ T cells producing inducible multiply spliced (ms)-Tat/rev transcript per million of CD4^+^ T cells was 4.54 in the morphine-administered group versus 9.11 in the saline control group (*P* = 0.067) (Fig. 5A). In the LNs, the median frequency of CD4^+^ T cells producing inducible ms-Tat/rev transcript per million of CD4^+^ T cells was 6.47 in the morphine-administered group versus 10.38 in the saline control group, which was statistically significant (*P* = 0.036) (Fig. 5B).

We next used the intact proviral DNA assay (IPDA) to estimate the size of replication-competent SIV reservoirs in both the blood and LNs. Recently Bruner et al. (46) developed a multiplex droplet digital PCR (ddPCR)-based HIV-1 proviral DNA quantification assay that can distinguish between intact versus defective proviruses and is referred to as IPDA. The authors have described a positive correlation and identical decay rate of viral reservoirs when measured by quantitative viral outgrowth assay (QVOA) and IPDA (46). Bender et al. have reported an adaptation of IPDA for quantification of the intact SIV genome in ART-suppressed rhesus macaques, implicating thereby its utility to serve as a surrogate marker to measure the size of latent reservoirs (47). Here, using IPDA, we measured the frequency of intact SIV genomes in both blood and LNs of morphine/saline-administered as well as SIV-infected cART-treated RMs (Fig. 1B). In CD4^+^ T cells purified from peripheral blood, the median number of intact SIV genomes per million cells was 1,480 for the morphine-administered group versus 2,521 in the saline control group (*P* = 0.080) (Fig. 5C). However, in the CD4^+^ T cells purified from LNs, the median intact SIV genomes per million cells was measured as 2,789 for the morphine-administered group versus 4,399 in the saline control group (*P* = 0.036) (Fig. 5D).

### Changes in CD4^+^ T cell polarization in PBMCs and lymph nodes.

**FIG 6:**
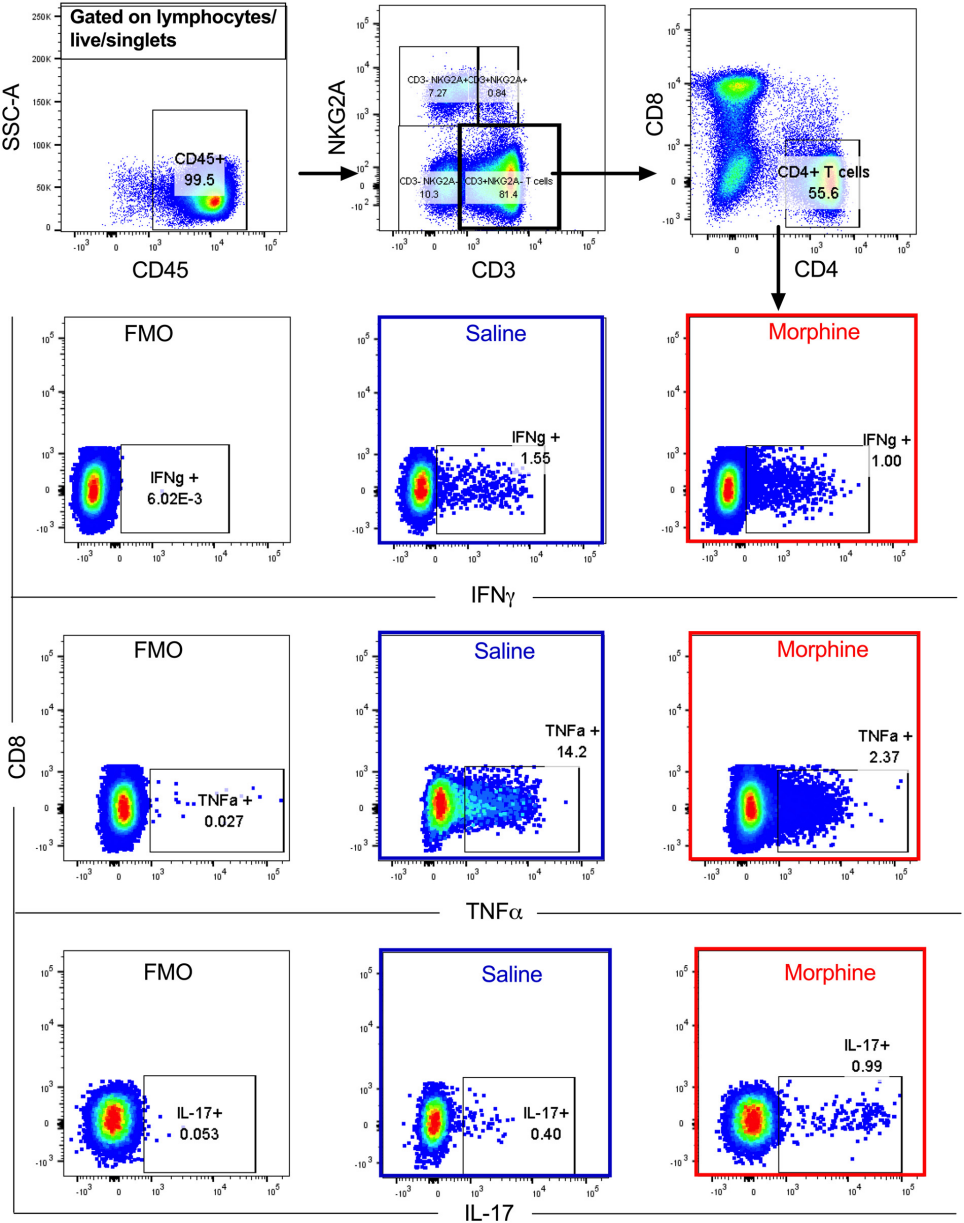
Gating strategy for CD4^+^ Th1/Th17 polarity in PBMCs. Briefly, CD45^+^ cells were gated and later NK T cells excluded from the total CD3^+^ T cell pool. CD4 and CD8 T cells were gated out of the total T cells. The CD4 T cells were then further investigated to determine the extent of IFN-γ, TNF-α, and IL-17 cytokine secretion following PMA and ionomycin stimulation.

**FIG 7:**
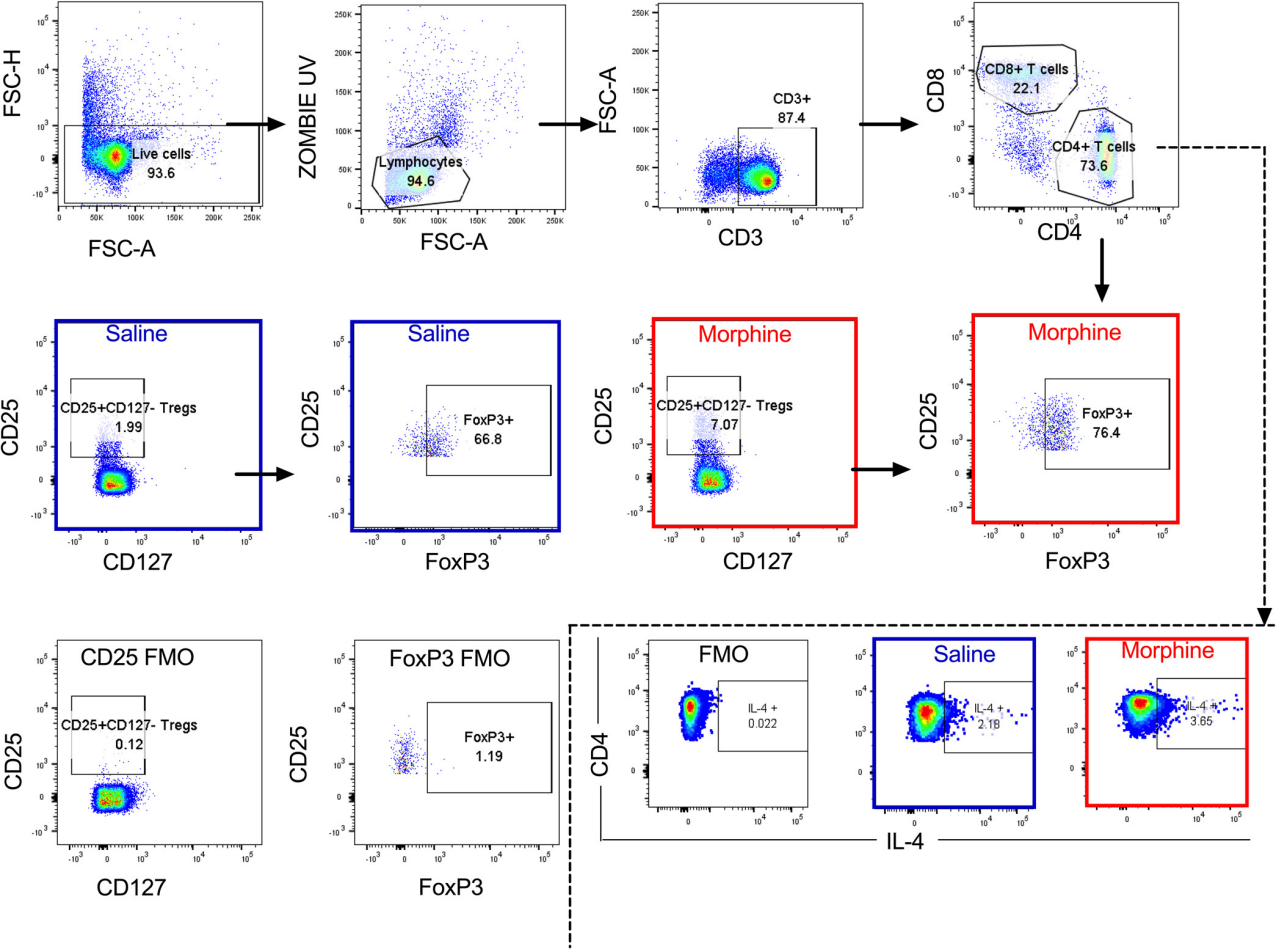
Gating strategy for Th2/T regs in PBMCs. Dead cells excluded based on zombie UV dye positive expression. Based on forward scatter area (FSC-A) versus side scatter area (SSC-A) gating, lymphocytes were later gated and CD4^+^ T cells further obtained from total CD3^+^ T cells. From the CD4^+^ T cells, the extent of IL-4 cytokine secretion was then evaluated following stimulation with PMA/ionomycin. Finally, the frequencies of CD4^+^ T regs were evaluated based on the expression of CD25 and absence of CD127. These cells were later evaluated for their expression of the transcription factor FoxP3 to give the final frequency of T regs denoted as percent CD4^+^ CD25^+^ CD127^−^ FoxP3^+^ cells.

Chronic morphine exposure depletes lymphoid cells and leads to a phenotypic switch of Th1 to Th2 in CD4^+^ T cells (38). On the other hand, a recent report suggests that in chronic HIV patients, the majority of intact replication-competent proviruses persist in Th1-polarized CD4^+^ T cells (48). To understand the impact of chronic morphine administration on T lymphocytes, flow cytometry was performed at necropsy to determine the CD4^+^ T cell polarity in the PBMCs and LNs of cART-treated RMs. In PBMCs, to differentiate between Th1, Th2, and Th17 cells, intracellular cytokine staining was performed as described earlier (49). Due to poor cytokine production by LN germinal center T follicular helper (Tfh) cells, it is difficult to differentiate Tfh subpopulations by cytokine production assays (50). Therefore, we performed surface staining of chemokine receptors of Tfh cells (CD95^+^ PD1^+^ CXCR5^+^) from LNs to identify Th1-like Tfh (CXCR3^+^), Th2-like Tfh (CCR4^+^), and Th17-like Tfh (CCR6^+^) subpopulations (51). The gating strategies used for PBMCs and LNs are described in Fig. 6, 7, and 8. In PBMCs, the frequency of CD4^+^ Th1 polarized cells was measured by quantifying levels of gamma interferon (IFN-γ) and tumor necrosis factor alpha (TNF-α) cytokine-secreting cells. In morphine-administered RMs, IFN-γ-secreting cells were a median of 0.87% versus 1.55% (*P* = 0.536) for controls (Fig. 9A), while TNF-α-secreting cells were a median of 5.61% versus 14.70% for controls, which was significantly different (*P* = 0.017) (Fig. 9B). The median of interleukin 4 (IL-4)-secreting Th2 polarized CD4^+^ T cells in the morphine-administered macaques was 2.55% versus 1.76% in saline controls (*P* = 0.305) (Fig. 9C). The levels of IL-17-secreting CD4^+^ Th17 lymphocytes in the morphine-treated group were a median of 0.47% versus 0.48% in saline controls (*P* = 0.930) (Fig. 9D). Remarkably, exposure to morphine in cART-treated SIV-infected RMs led to a significant elevation of CD4^+^ Treg lymphocytes, with a median of 5.22% versus 2.95% in the saline control group (*P* = 0.004) (Fig. 9E).

**FIG 8:**
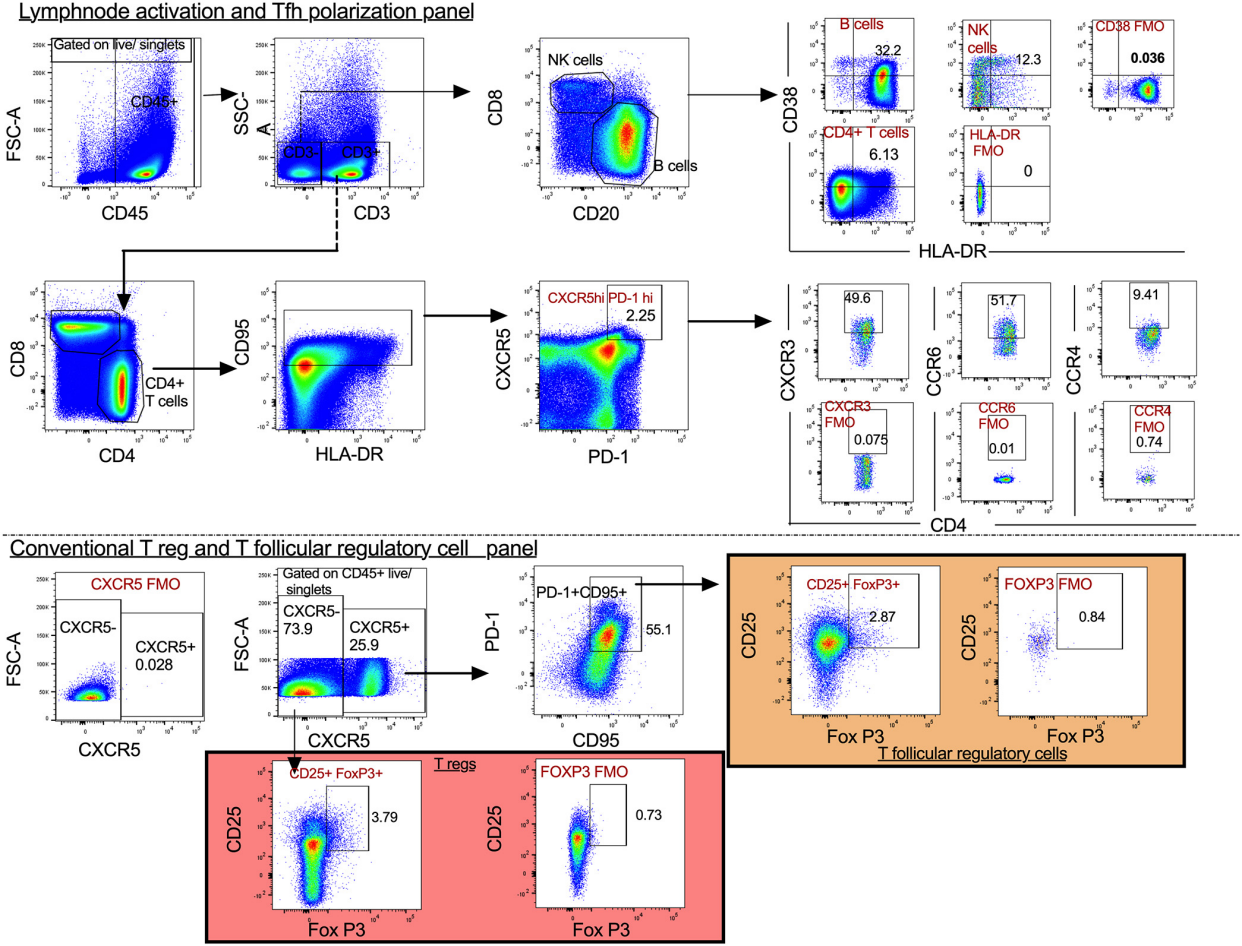
Gating strategy for lymph node activation and Tfh polarization panel. CD45^+^ leukocytes were gated and T and non-T lymphocytes discriminated based on CD3 expression. CD3^−^ cells were segregated based on those that were CD20^+^ (B cells) and CD8α^+^ (NK cells). The activation profiles of these cells were further evaluated based on CD38 and HLA-DR coexpression. CD4^+^ T cells were separately gated out of CD3^+^ T cells and levels of activation determined. The CD95^+^ memory cell marker was then included to obtain memory CD4^+^ T cells and later delineate the Tfh population based on CXCR5 and PD-1. Additional chemokines were used to study Tfh polarity based on Tfh1 (CXCR3), Tfh2 (CCR4), Tfh17 (CCR6), and T follicular regulatory cells (CD25 and FoxP3).

**FIG 9:**
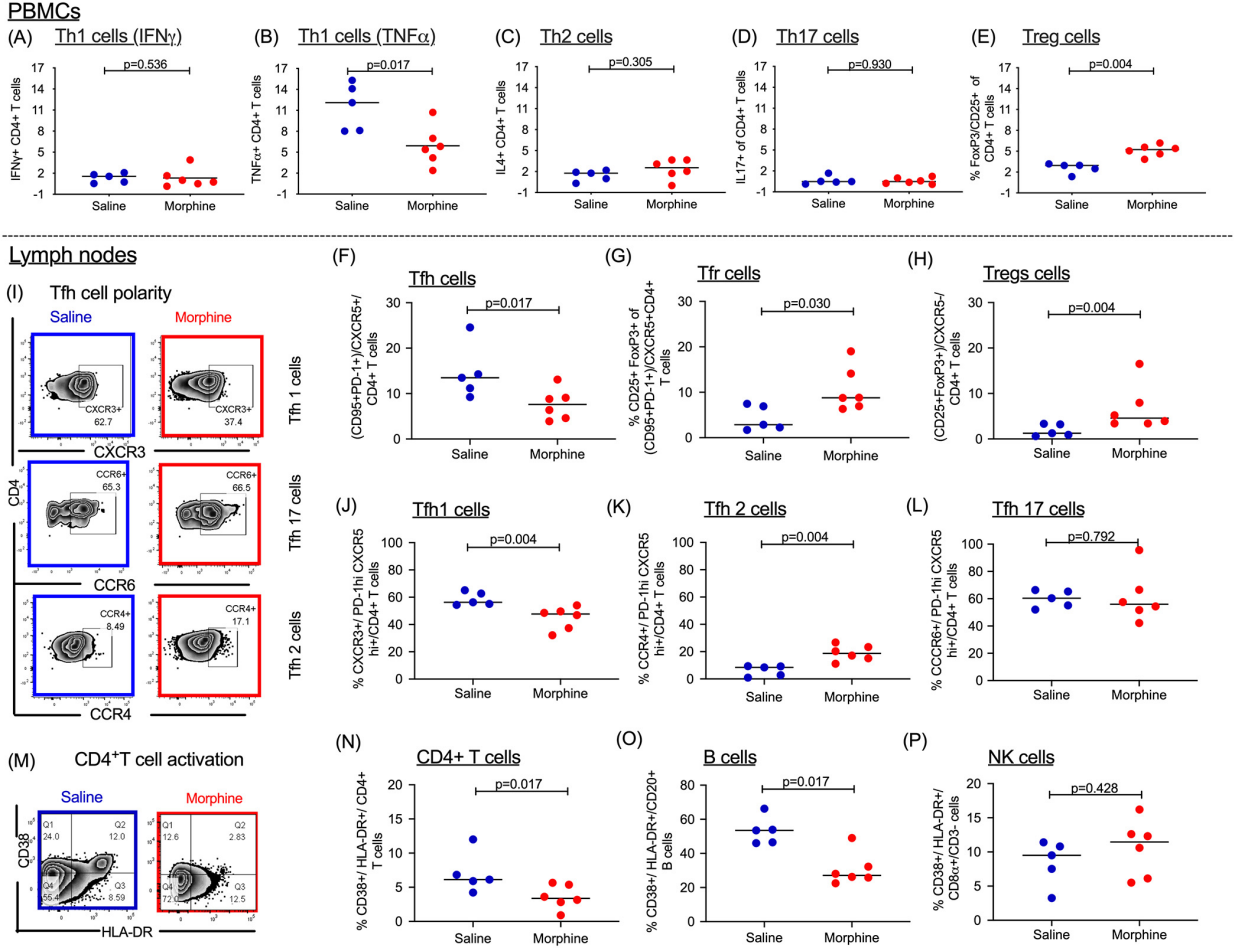
Immune dynamics in peripheral blood and lymph node compartments in morphine-administered versus saline (control) groups of cART-treated SIVmac251-infected rhesus macaques. Following stimulation with PMA/ionomycin, differences in the levels of PBMC CD4^+^ Th1 cells secreting IFN-γ (A), PBMC CD4^+^ Th1 cells secreting TNF-α (B), PBMC CD4^+^ Th2 cells secreting IL-4 (C), PBMC CD4^+^ Th17 cells secreting IL-17 (D), and PBMC Tregs (E) were also outlined as CD25^+^ FoxP3^+^ CD4^+^ T cells. (F) Within the lymph nodes, the frequencies of T follicular helper (Tfh) cells based on CD95 and PD-1 coexpression among CXCR5^+^ T cells were assessed. (G) Similarly, the levels of T follicular regulatory cells (Tfr) were evaluated based on CD25 and FoxP3 coexpression among the Tfh population. (H) Simultaneous evaluation of T regs (CD25^+^ and FoxP3^+^) within the CXCR5^−^ CD4^+^ T cell population was also carried out. (I) Representation of Tfh polarity as delineated as Tfh1, Tfh 17, and Tfh 2 based on the surface expression of specific chemokine receptors. Collectively, Tfh1 was based on CXCR3 (J), Tfh17 based on extent of CCR6 (K), and Tfh2 based on CCR4 expression (L) among CXCR5^hi^ PD-1^hi^ Tfh cells. (M) Representation of CD4^+^ T cell activation in one experimental and one control sample. Levels of CD4^+^ T cell (N), B cell (O), and NK cell (P) activation as measured by the extent of CD38 and HLA-DR coexpression.

Within the LNs, morphine-administered cART-treated RMs had lower frequencies of T follicular helper cells (Tfh) expressing PD-1, CXCR5, and CD95, with a median value of 7.61% versus 13.49% in saline control groups (*P* = 0.017) (Fig. 9F). On the other hand, in the morphine-administered group, the frequency of regulatory Tfh (CD25^+^ FoxP3^+^ Tfh) cells was elevated, with a median of 8.78% versus 2.87% in saline control group (*P* = 0.030) (Fig. 9G). Similarly, there was an elevation of CD4^+^ CXCR5^−^ Treg (CD25^+^ FoxP3^+^) cells in the morphine-administered group, with a median of 4.57% versus 1.25% in saline control group (*P* = 0.004) (Fig. 9H). Evaluation of Tfh cell polarity was also carried out as shown by the representative sample in Fig. 9I. There were differences in Tfh cell differentiation (Th1 versus Th2) in the morphine-administered group versus that in the saline controls. In the morphine group, while there was a reduction in CXCR3^+^ Tfh cells (Th1), with a median of 47.70% versus 56.30% in the saline control group (*P* = 0.004) (Fig. 9J), there was an elevation in CCR4^+^ Tfh cells (Th2), with a median of 18.70% in the morphine-administered group versus 8.49% in the saline control group (*P* = 0.004) (Fig. 9K). Furthermore, CCR6^+^ Tfh cells (Th17) were a median of 55.95% in the morphine-administered RMs versus 60.40% in saline control RMs (*P* = 0.792) (Fig. 9L). Finally, differences in immune activation across different cell subsets were evaluated. Figure 9M denotes a representative sample for CD4^+^ T cell activation. For CD4^+^ T cell activation (denoted by CD4^+^ CD38^+^ HLA-DR^+^), the morphine-administered RMs had a significantly lower frequency, with a median of 3.37% versus 6.13% in saline controls (*P* = 0.017) (Fig. 9N). Similarly, for B cell activation (denoted by CD20^+^ CD38^+^ HLA-DR^+^), the morphine-administered macaques had a significantly reduced frequency, with a median of 27.10% versus 53.50% in saline controls (*P* = 0.017) (Fig. 9O). On the other hand, for NK cell activation (CD3^−^ CD8α/CD38^+^ HLA-DR^+^), the morphine-administered group had a median frequency of 11.45% versus 9.16% in the saline control group (*P* = 0.428) (Fig. 9P).

### Quantification of SIV reservoirs in the CNS.

**FIG 10:**
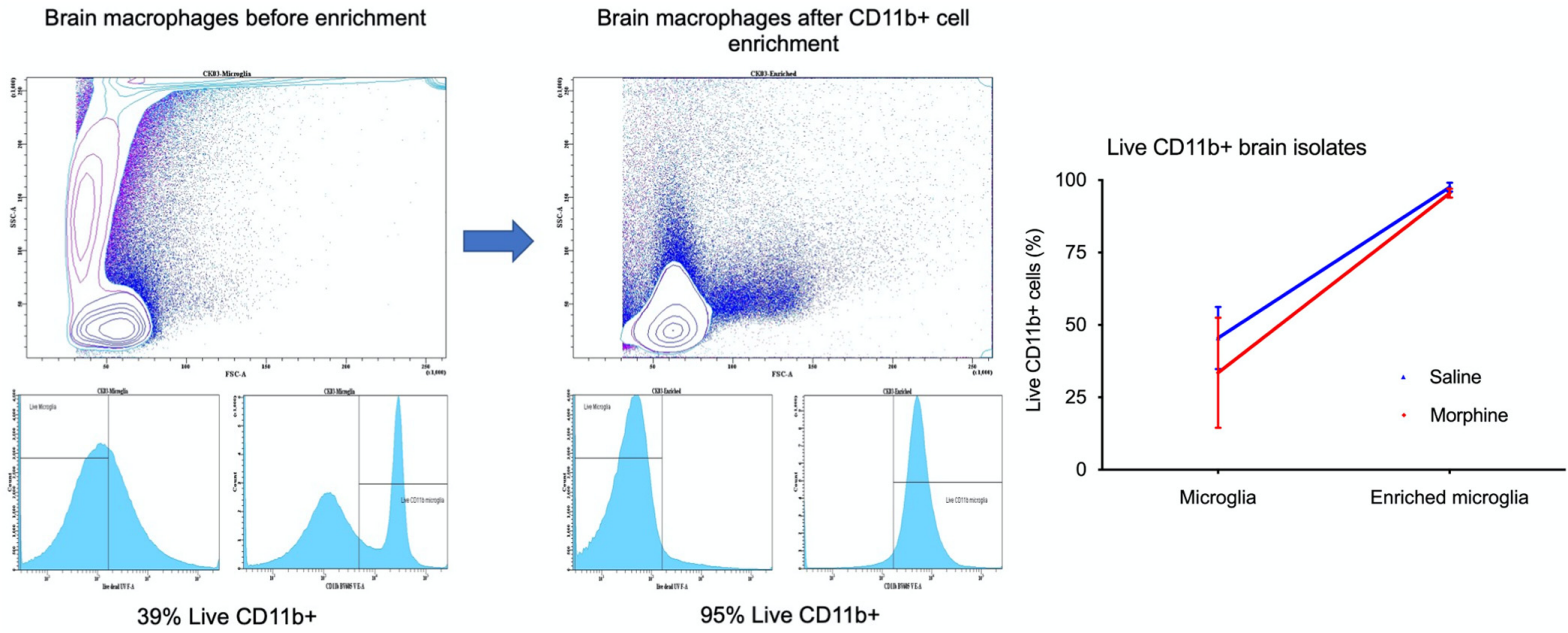
Gating strategy to detect the purity of CD11b^+^ macrophages isolated from brains of rhesus macaques. Briefly, comparisons in the subsequent purity of CD11b microglia showed that higher yields of brain macrophages were obtained following enrichment of brain cells with CD11b beads.

**FIG 11:**
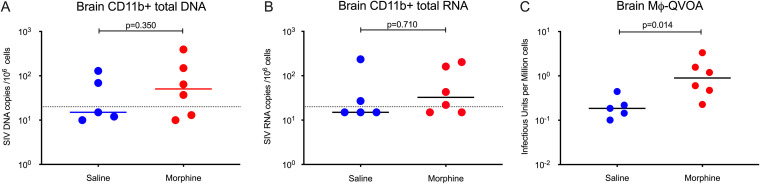
Sizes of SIV reservoirs in CD11b^+^ macrophages from brains of morphine-administered versus control groups of SIVmac251-infected ART-suppressed rhesus macaques. (A) SIV DNA copies per million CD11b^+^ macrophages isolated from brains of morphine-administered and control rhesus macaques that received saline. (B) SIV RNA copies per million CD11b^+^ macrophages isolated from brains of morphine-administered and control rhesus macaques that received saline. (C) Sizes of functional latent reservoirs estimated by macrophage quantitative viral outgrowth assay (MØ-QVOA) in CD11b^+^ macrophages purified from brains of morphine-administered and control rhesus macaques that received saline. Infectious unites per million cells (IUPM) were estimated using the IUPMStats v1.0 infection frequency calculator. All values below the limit of detection were brought to the limit of detection for better representation in the figure.

To understand how chronic morphine administration impacted the seeding and persistence of viral reservoirs in the CNS, we analyzed CD11b^+^ myeloid cells (microglia and perivascular macrophages) from the brains of the cART-treated morphine-administered and saline control SIV-infected monkeys, all of which had complete viral suppression in the plasma and CSF (<50 copies/ml). Immediately after necropsy, CD11b^+^ cells were purified from brains. After purification, we obtained more than 95% cells positive for CD11b (Fig. 10). The median of cell-associated DNA load was 50.5 copies per million CD11b^+^ microglia/macrophages in the morphine-administered group versus 0 in the saline control group (*P* = 0.350) (Fig. 11A). The median cell-associated RNA load was 32.5 copies per million CD11b^+^ microglia/macrophages in the morphine-administered group versus 15 in the saline control group (*P* = 0.710) (Fig. 11B). We next sought to estimate the size of latent replication-competent SIV reservoirs in CD11b^+^ microglia/macrophages using macrophage QVOA (MØ-QVOA). The number of CD11b^+^ macrophages used for the MØ-QVOA for each animal is described in Table 1. On average, we detected 0.075% CD3^+^ T cells within the enriched CD11b^+^ cells from brain, which is in line with 0.06% reported by Avalos et al. (52). Based on percentage of CD4^+^ T cells of the total CD3^+^ T cells and the frequency of CD4^+^ T cells having intact proviral genome (as determined by IPDA), the probability of the presence of CD4^+^ T cells with intact proviral genome in any sample of macrophage quantitative outgrowth assay was found to be less than 1. The CD11b^+^ microglia/macrophages obtained from morphine-administered macaques had a median infectious unit per million (IUPM) value of 0.89 versus 0.19 in saline control group (*P* = 0.014) (Fig. 11C), indicating the presence of a significantly higher number of cells carrying infectious SIV in the morphine group.

**TABLE 1:** Number of brain macrophages and percentage of contaminating CD3^+^ T cells in the MØ-QVOA for each animal and corresponding IUPM values

Serial no.	Animal ID^*a*^	% of contaminating CD3^+^ T cells in CD11b^+^ cells by TCRβ RNA level	Probability of presence of CD4^+^ T cells with intact proviral genome in MØ-QVOA	No. of cells used in MØ-QVOA	IUPM for CD11b^+^ macrophages
1	CK03	0.071	<1	1.67E+07	0.185
2	CK07	0.079	<1	1.00E+07	0.101
3	CK40	0.072	<1	1.67E+07	0.144
4	CI04	0.190	<1	1.67E+07	0.219
5	CI47	0.118	<1	3.33E+07	0.444
6	CK22	0.034	<1	2.33E+06	0.602
7	CK31	0.020	<1	1.00E+07	1.194
8	CK48	0.015	<1	3.33E+06	3.330
9	CH89	0.019	<1	1.67E+07	1.569
10	CI83	0.205	<1	1.67E+07	0.473
11	CI90	0.003	<1	1.67E+07	0.227

a ID, identifier.

## DISCUSSION

In order to achieve a functional cure of HIV-1, the primary goal is to identify all cell types and anatomical sanctuaries of viral reservoirs and to understand the molecular mechanism of their long-term persistence. Substantial work has already been done to characterize and understand the underlying mechanism of latent reservoirs in circulating CD4^+^ T cells and in lymphoid tissues. Several lines of evidence suggest that within 3 to 7 days of infection, HIV enters the CNS as well as tissue resident macrophages and microglia, which likely serve as viral reservoirs and are a source of viral rebound in the cases of therapeutic interruptions (34, 53–55). The mechanism of persistence of HIV reservoirs in myeloid cells is still unknown (35). Substance use disorders (SUD) and, more specifically, OUD are an important comorbidity among PLWH (23). Opioids have immune modulatory effects and mostly render macrophages more permissive to HIV infection (21, 24, 56, 57). However, the impact of opioids on seeding and persistence of HIV reservoirs remains underscored, and this gap poses a major roadblock in HIV cure research. To shed some light on this direction, in the present study, we used SIVmac251-infected rhesus macaques as a nonhuman primate (NHP) model of HIV infection to understand how opioid use could modulate the size of viral reservoirs. We chose the SIVmac251 stock for intravenous infection since it contains multiple quasispecies of various tropism (58), and intravenous drug users (IVDU) are often initially infected by multiple transmitted viral sequences (59). Recently, Abreu et al. showed that, indeed, ART-suppressed SIVmac251 rhesus macaques are an ideal model for studying the viral reservoirs from different anatomical tissue sanctuaries (60).

The results of the present study indicate there was no difference in the geometric means of plasma and CSF viral loads between morphine-administered and control animals receiving saline in both untreated and ART-suppressed groups. However, in a previous study, Bokhari et al. (18) reported that morphine-administered rhesus macaques exhibited 1-log higher plasma and CSF viral loads than controls. It should be noted that there are a couple of differences between the study of Bokhari et al. (18) and the present study. Bokhari et al. (18) administered morphine in four equal doses daily, whereas we administered two equal daily doses. Since the plasma half-life of morphine is around 2.5 h (61), the difference in dosage could lead to variances in the mean effective plasma concentration of the drug. Furthermore, while we used SIVmac251, Bokhari et al. (18) used a brain-derived stock of SIV (SIVmacR71/17E). In the Bokhari et al. (18) study, a high rate of rapid progression of disease was found in the morphine-treated group, and similar findings were obtained by Kumar et al. using a mixture of different SIVs [SHIV(KU), SHIV(89.6)P, and SIV/17E-Fr] (62). Furthermore, as the rhesus macaques used in different studies are outbred from multiple colonies, it is expected that there would be a wide variation in their genetic backgrounds and, subsequently, their responses to morphine administration and SIV disease pathogenesis. These differences could have contributed to the differences of plasma and CSF viral loads observed in these studies.

Among the untreated RMs, we did not find any difference in cell-associated DNA and RNA loads at different tissue compartments between morphine-administered and saline control groups. On the other hand, in cART-suppressed RMs, we observed a significant reduction in cell-associated DNA load in CD4^+^ T cells obtained from LNs as well as in tissue samples collected from rectal mucosa in morphine-administered macaques compared to that in saline controls. This finding is substantiated by IPDA and TILDA performed on CD4^+^ T cells purified from LNs, where the number of intact SIV genomes per million CD4^+^ T cells was less and the size of inducible SIV reservoirs in CD4^+^ T cells was lowered significantly in morphine-administered RMs compared to that in saline controls, respectively. These findings indicate that chronic morphine administration in combination with ART has a positive impact on lowering SIV reservoirs in lymphoid tissues.

The CD4^+^ T cells are a major source of latent reservoirs of HIV in lymphoid tissues such as LNs, GALT, and in peripheral blood, among others (63). In LNs, HIV reservoirs seeded during acute infection are associated with virus production and storage of viral particles in immune complexes, and Tfh cells constitute the major part of LN viral reservoirs (64–66). We observed depletions of Tfh cells in LNs of morphine-administered macaques that support earlier reports that chronic morphine abuse depletes the volume and total number of lymphoid cells from LNs (67, 68). Brown et al. reported that morphine induces an immunosuppressive effect in LNs and peripheral blood of African green monkeys and pigtailed macaques, with a substantial decrease in the abundance of several metabolic proteins involved in energy metabolism pathways accompanied by significant decreases in activated CD4^+^/CD8^+^ T cells (69). Therefore, we speculate that in our experimental setting, chronic morphine administration may dampen the T cell activation, suppress their metabolic activity, and cause a reduction in seeding of SIV in CD4^+^ T cells during acute stage of infection before initiation of cART, which may result in a smaller size of persistent latent SIV reservoirs in CD4^+^ T cells of morphine-administered animals than in control macaques that received saline.

**FIG 12:**
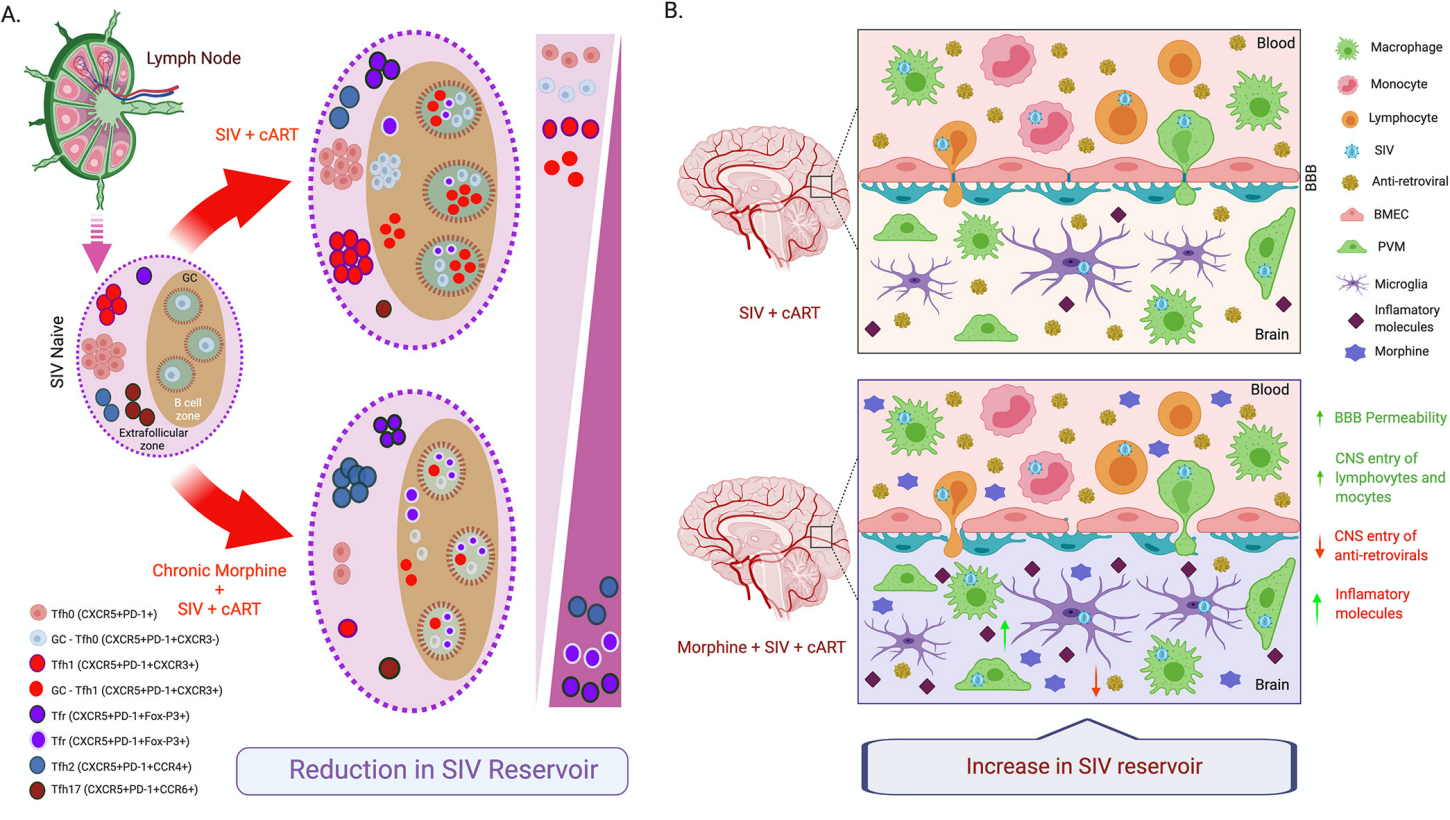
Proposed mechanism of morphine-mediated differential regulation of SIV reservoirs in lymphoid tissue versus that in myeloid cells in the CNS. (A) Chronic morphine exposure leads to a reduction of Tfh cells in lymph nodes (LNs), which serve as the major HIV/SIV reservoirs in LNs. This may be responsible for the observed reduction in SIV reservoirs in our morphine-dependent, SIVmac251-infected cART-treated rhesus macaque model. (B) Chronic morphine exposure increases the permeability of the blood brain barrier (BBB), which increases the trafficking of lymphocytes and monocytes to the CNS. Again, opioids lower the CNS penetration of several antiretroviral drugs to facilitate the basal level of ongoing HIV replication in brain. This may be responsible for the larger size of functional SIV reservoirs in our morphine-dependent, SIVmac251-infected cART-treated rhesus macaque model.

Next, we observed that morphine administration resulted in the expansion of regulatory T cells in peripheral blood as well as in LNs, which have been previously reported to suppress T-cell activation (70). In LNs, we also observed lower level of activation of CD4^+^ T cells and B cells in morphine-administered animals than in saline controls. This supports the proposed mechanism of morphine-mediated reduction of SIV reservoirs within the lymphoid tissue compartments. In the LNs of morphine-administered animals, we observed a reduction in frequency of CXCR3^+^ Th1-like Tfh cells and expansion of CCR4^+^ Th2-like Tfh cells, and it was reported earlier that Th1-like Tfh cells enter the LN germinal center during the acute stage of HIV/SIV infection, express a high level of CCR5, and constitute a major part of viral reservoirs in LNs (50, 71, 72). In combination, the findings as described above may lead to a depletion of functional SIV reservoirs in CD4^+^ T cells of morphine-administered ART-suppressed rhesus macaques (Fig. 12A).

Contrary to the CD4^+^ T cells, we observed an opposite effect of morphine in modulation of the viral reservoir in the CNS. The median size of replication-competent SIV reservoirs in CD11b^+^ microglia/macrophages from the brain was significantly larger in morphine-administered RMs than in control animals receiving saline. There are several factors that may be responsible for the morphine-mediated modulation of CNS SIV reservoirs. It has been reported that morphine upregulates the expression of CCR5 coreceptors in macrophages, which, in turn, could increase the viral permissiveness of these cells (21, 57, 73). Morphine is known to also lower the CNS penetration of several antiretrovirals, which could result in ongoing viral replication in the CNS and be responsible for the larger reservoir (74). Again, it has been shown that morphine alone or in combination with HIV Tat increases the permeability of the blood brain barrier (BBB) and increases transendothelial migration of leucocytes by activation of proinflammatory cytokines, intracellular Ca^2+^ release, and activation of myosin light chain kinase that downregulate tight junction proteins and decrease transendothelial electric resistance (26, 75, 76). It also increases the release of CCL2, CCL5, and IL-6 by astrocytes and upregulates expression of ICAM and VCAM on brain vascular endothelial cells that promote trafficking of HIV-infected peripheral leucocytes in the CNS (77). Morphine is also shown to downregulate the expression of anti-HIV microRNAs and impair the function of anti-HIV restriction factors in monocytes (78, 79). Taken together, these could lead to increased size of the viral reservoirs in the CNS of the morphine-administered group in comparison to that in the controls that received saline (Fig. 12B). We observed a similar analogy with our finding of differential modulation of SIV reservoirs by morphine in T cells from PBMC/LNs versus from microglia/macrophages in the CNS in some previous reports where phenelzine, a monoamine oxidase (MAO) inhibitor, suppressed reactivation of HIV in T cells (80) and reactivated HIV in human microglia cell lines (81). To the best of our knowledge, this is the first report of opioid-mediated differential modulation of HIV reservoirs using macaque models of HIV infection.

The major caveat of the present study is the number of RMs included in each group due to the prohibitive cost of conducting long-term studies with multiple daily injections. Other issues include the experimental design, in which animals were initially ramped-up with morphine, then infected, and then treated with cART. In a patient cohort, this may not be an ideal scenario, as most of the people suffering from substance use disorders take multiple forms of drugs. Furthermore, opioid use may follow infection; for example, in PLWH, opioids are prescribed to treat chronic pain, and subsequently they may become addicted to these substances. For this population, how opioid use modulates the dynamics of different viral reservoirs needs to be explored. In addition, given the different cellular nature of the reservoir (CD4^+^ T cells in lymphoid tissues and macrophages in the CNS), the possible effects of opioids on viral tropism is unknown.

In conclusion, for the first time, we describe a morphine-dependent SIVmac251-infected RM model to study the impact of opioid use disorder (OUD) on HIV reservoirs. Our results suggest that morphine differentially modulates SIV reservoirs in LNs and rectal mucosal tissue versus SIV reservoirs in myeloid lineage of cells within the CNS. Chronic morphine administration reduces the size of viral reservoirs in CD4^+^ T cells in LNs and increases the size of SIV reservoirs in brain-resident CD11b^+^ macrophages. We propose that these preclinical models will serve as a tool to discover the molecular mechanism of opioid-mediated differential regulation of viral reservoirs among PLWH and suffering from OUD.

## MATERIALS AND METHODS

### Reagents and cell lines.

Antiretroviral drugs tenofovir alafenamide (TFV) and emtricitabine (FTC) were obtained from Gilead Sciences, Foster City, CA, USA, while dolutegravir (DTG) was procured from ViiV Healthcare, Research Triangle Park, NC, USA, as a material transfer agreement (MTA) with S. N. Byrareddy. All other molecular biology-grade fine chemicals used in the study were purchased from Sigma-Aldrich, St. Louis, MO, USA, unless otherwise mentioned. CEMx174 is a hybrid human lymphoid cell line generated from human B721.174 and T-CEM cell lines that was used for expansion of virus in quantitative viral outgrowth assays due to its enhanced susceptibility to SIV infection (82). This cell line was provided by J. Hoxie (University of Pennsylvania, Philadelphia, PA). CEMx174 cells were maintained in complete RPMI (RPMI 1640) medium (Gibco; cat. no. 21870076) with 10% heat-inactivated fetal bovine serum (FBS) (Gibco; cat. no. 10437-028), 2 mM l-glutamine (Gibco; cat. no. 35050079), and 100 U/ml penicillin and 100 μg/ml streptomycin (Gibco; cat. no. 15140-122) at 37°C and 5% CO_2_.

### Animals and ethical statement.

A total of 19 Indian-origin, outbred pathogen-free RMs (Macaca mulatta; mean age, 4.67 years; range, 4.1 to 7.0 years) were used in this study (Table 2). Macaques were housed in compliance with the regulations under the Animal Welfare Act, the Guide for the Care and Use of Laboratory Animals in the nonhuman primate facilities at the Department of Comparative Medicine, University of Nebraska Medical Center (UNMC), Omaha, NE, USA. Animals were maintained in a temperature-controlled (72°F) indoor climate with 12-h light/dark cycle. The monkeys were observed twice daily for development of distress or disease by the animal care staffs and veterinary personnel. The animals were daily fed monkey diet (Purina) supplemented with fresh fruit or vegetables and water *ad libitum*.

**TABLE 2:** Descriptions of rhesus macaques used in the study

Serial no.	Animal ID^*a*^	Age (yrs)	*Mamu* genotype			Treatment group	Antiretroviral therapy^*b*^	Plasma viral load (copies/ml)		CSF viral load (copies/ml)	
			*A01	*B08	*B17			Peak	At necropsy	Peak	At necropsy
1	12N067	7	+	−	−	Saline	None	9.02E+06	4.24E+04	1.38E+05	1.19E+04
2	15N223	5	−	−	−	Saline	None	7.22E+06	1.04E+05	1.82E+04	7.37E+03
3	14T014	5	−	−	−	Saline	None	1.89E+07	3.22E+08	6.49E+04	1.81E+04
4	14X006	5.2	−	−	−	Saline	None	3.37E+07	5.11E+08	2.45E+04	2.57E+05
5	CI47	4.2	−	+	−	Saline	TFV+FTC+DTG	2.31E+07	<LOD^*c*^	3.60E+03	<LOD
6	CK03	4.3	−	−	−	Saline	TFV+FTC+DTG	9.87E+06	<LOD	1.64E+05	<LOD
7	CI04	4.4	−	−	−	Saline	TFV+FTC+DTG	8.69E+06	<LOD	3.48E+05	<LOD
8	CK07	4.4	−	−	−	Saline	TFV+FTC+DTG	1.83E+07	<LOD	2.03E+05	<LOD
9	CK40	4.2	−	−	−	Saline	TFV+FTC+DTG	1.25E+07	<LOD	1.03E+05	<LOD
10	14X002	5	−	−	−	Morphine	None	8.68E+07	1.80E+06	1.13E+05	9.25E+04
11	13T005	5.3	NA^*d*^	NA	NA	Morphine	None	1.85E+07	3.05E+06	4.51E+06	5.21E+02
12	14X043	5	−	−	+	Morphine	None	8.10E+05	3.18E+04	9.97E+03	4.12E+03
13	15N012	4.4	−	−	−	Morphine	None	1.02E+07	3.07E+05	1.22E+05	1.27E+02
14	CK31	4.1	+	−	−	Morphine	TFV+FTC+DTG	2.00E+07	<LOD	7.85E+02	<LOD
15	CK22	4.2	−	+	−	Morphine	TFV+FTC+DTG	4.25E+06	<LOD	1.91E+03	<LOD
16	CK48	4.2	+	−	−	Morphine	TFV+FTC+DTG	3.92E+06	<LOD	3.08E+05	<LOD
17	CI83	4.3	−	−	−	Morphine	TFV+FTC+DTG	1.20E+07	<LOD	1.05E+04	<LOD
18	CI90	4.2	−	−	−	Morphine	TFV+FTC+DTG	1.07E+06	<LOD	9.62E+03	<LOD
19	CH89	4.4	−	−	−	Morphine	TFV+FTC+DTG	1.81E+07	<LOD	7.47E+04	<LOD

a ID, identifier. All animals were male.

b TFV, tenofovir alafenamide; FTC, emtricitabine; DTG, dolutegravir.

c LOD, limit of detection.

d NA, not available.

At the end of the study, all RMs were humanely euthanized using a high dose of ketamine-xylazine, and then the thoracic cavity was opened and perfused/exsanguinated according to the guidelines of the American Veterinary Medical Association. This study was reviewed and approved by UNMC Institutional Animal Care and Use Committee (IACUC) and the Institutional biosafety Committee (IBC) under protocol number 16-073-07-FC titled “The effect of cART and drug of abuse on the establishment of CNS viral reservoirs” and 15-113-01-FC titled “The combinatorial effects of opiates and promoter-variant strains of HIV-1 subtype C on neuropathogenesis and latency.” UNMC has been accredited by the Association for Assessment and Accreditation of Laboratory Animal Care International.

### Study design.

The overall design of the study is illustrated in a schematic form in Fig. 1. These experiments were designed to investigate the effect of chronic morphine administration on the establishment of viral reservoir in SIV-infected RMs in different anatomical compartments of the body. The study included 19 juvenile RMs. The RMs were randomly divided into two groups. One group had 10 animals which were ramped-up over 2 weeks to a final 6-mg/kg intramuscular injection of morphine administered twice daily, which was then maintained for 7 weeks, and the other group (*n* = 9) received a similar dose of normal saline (control group). At this point, all the macaques were intravenously inoculated with 200 TCID_50_ of SIVmac251 (viral stock was obtained from Mahesh Mohan from Tulane National Primate Research Center), while the administration of morphine/saline continued until the end of the study. At 5 weeks postinoculation, daily ART was initiated in six macaques from the morphine group and five macaques in the control group and continued until the end of the study. ART regimen consisted of two reverse transcriptase inhibitors (FTC, 40 mg/ml, and TFV, 20 mg/ml) and one integrase inhibitor (DTG, 2.5 mg/ml). All the drugs were dissolved in a vehicle made up of 15% Kleptose HPB (Roquette, parenteral grade) (wt/wt) in 0.1 N NaOH. The antiretroviral drugs were administered subcutaneously once daily at 1 ml/kg body weight. Peripheral blood from the femoral vein, CSF by direct puncture of the cisterna magna or by lumbar puncture, LNs, and colorectal mucosa biopsy specimens were collected longitudinally at different time points of the study after anesthetizing the monkeys with ketamine-HCl (5 to 20 mg/kg) or tiletamine and zolazepam (Telazol, 3 to 5 mg/kg) to monitor SIV viral loads and a series of immunologic and virologic parameters as described in the experimental schema.

### SIV plasma and CSF viral load quantification.

SIV RNA concentration in the plasma and CSF samples were measured by quantitative reverse transcription-PCR (qRT-PCR) as previously described (83). In brief, plasma was separated from blood samples collected in K2-EDTA vacutainer tubes (Becton, Dickinson, San Diego, CA, USA) within 4 h of collection. RNA was extracted from 140 µl of plasma and CSF samples using a QIAamp viral RNA minikit according to the manufacturer’s instructions (Qiagen, Germantown, MD, USA; cat. no. 52906). SIV gag RNA was quantified by qRT-PCR using the TaqMan RNA-to-Ct 1-Step kit (Thermo Fisher Scientific, MA; cat. no. 4392938) and Applied Biosystems QuantStudio 3 real-time PCR system (Applied Biosystems, Waltham, MA, USA). Primers and probes used for SIV gag RNA quantification were as follows: SIVGAGF, 5′-GTCTGCGTCATCTGGTGCATTC-3′; SIVGAGR, 5′-CACTAGGTGTCTCTGCACTATCTGTTTTG-3′; and SIVP, 5′-/6-carboxyfluorescein (FAM)/CTTCCTCAG/ZEN/TGTGTTTCACTTTCTCTTCTGCG/3IABkFQ-3′.

### Purification of CD4^+^ T cells.

Peripheral blood mononuclear cells (PBMCs) and LN cells were enriched for CD4^+^ T cells using the EasySep NHP CD4^+^ T cell isolation kit from STEMCELL Technologies Canada Inc. (cat. no. 19582) as per the manufacturer’s instructions. Briefly, frozen cells were thawed at 37°C, washed with complete RPMI (composition described above), centrifuged for 6 min at 1,200 rpm, and resuspended in 1 ml of recommended medium (phosphate-buffered saline [PBS] containing 2% fetal bovine serum [FBS] and 1 mM EDTA). The cell suspension was transferred to a 12-by-75-mm polystyrene tube, 50 µl of EasySep negative selection cocktail was added and mixed well, and the cell suspension was incubated at room temperature (RT) for 10 min. Then 100 µl of EasySep magnetic particles were added, and the cells were incubated at RT for 5 min. After incubation, 2 ml of recommended medium was added to the solution, and then the tubes were placed in an EasySep magnet for 5 min. The negatively selected enriched CD4^+^ T cells were collected for downstream applications.

### Isolation of total myeloid-enriched brain cells.

Myeloid-enriched brain isolation used a modification of the procedure described by Marcondes et al. (84). In brief, the brain was sectioned and meninges removed in Hanks’ balanced salt solution (HBSS; Invitrogen, Carlsbad, CA). The cleaned tissue was homogenized with a dounce homogenizer and washed twice with HBSS. Brain homogenate was digested on a rotating platform at 37°C for 30 min in HBSS with 28 U/ml DNase1 and 8 U/ml papain (Sigma, St. Louis, MO). The samples were triturated after 15- and 30-min digestion. Enzymes were inactivated with addiion of 3% FBS. Sample was washed twice with HBSS and then spun for 15 min at 1,800 rpm at 4°C through 25% Percoll (GE HealthCare, Pittsburg, PA). The resulting fatty upper and fluid middle layers were removed from the pellet. The pellet was resuspended in 10 ml of ice-cold HBSS and filtered through a 40-µm screen. Brain isolates were counted on a hemocytometer, and viability was measured by Trypan blue exclusion.

### Enrichment of CD11b myeloid cells.

Cell isolates were washed in PBS, and reconsituted in MACS buffer with 0.1% bovine serum albumin (BSA) (Miltenyi, Gladbach, Germany). Cells were counted and the volume was adjusted for staining with nonhuman primate CD11b microbeads (Miltenyi). Forty million cells were reconstituted in 160 µl of MACS buffer and reacted with 80 µl of CD11b microbeads at 4°C for 15 min. After incubation, cells were washed with MACS buffer with 0.1% BSA, reconstituted into 500 µl of MACS buffer, and loaded onto MACS Separator LD columns. Both the negative flowthrough and the positve CD11b^+^ cells were collected, and cells were counted on Coulter Counter Z1. Isolates were used for downstream processing and analyzed for epitope expression using a fluorescence-activated cell sorter (FACS).

### Purification of alveolar macrophages from bronchoalveolar lavage fluid.

At necropsy, the lung was lavaged with sterile saline, and the bronchoalveolar lavage (BAL) fluid was collected. Cells were collected through centrifugation of the BAL fluid at 250 × *g* for 10 min at 4°C. The cell pellets were washed twice with PBS containing 2% FBS and 2 mM EDTA. Cells were resuspended in complete RPMI (composition described above) and used for downstream applications.

### Isolation DNA/RNA from CD4^+^/CD11b^+^ cells.

Enriched CD4^+^ T cells and CD11b^+^ macrophages were pelleted by centrifugation for 6 min at 1,200 rpm and stored at −80°C until time of processing. To extract the DNA/RNA from the cells, first, the pellets were thawed on ice and thoroughly loosened by flicking the tube. Then, 600 µl Buffer RLT Plus with 2-mercaptoethanol (β-ME) at 10 µl/1 ml concentration was added and thoroughly mixed by vortexing. Following lysis, the mixture was transferred to a QIAshredder (Qiagen, cat. no. 79656), and the AllPrep DNA/RNA minikit (Qiagen, cat. no. 80204) was used for DNA/RNA isolation. The remaining protocol followed was per the kit’s instructions. RNA was eluted in 30 µl of RNase-free water after performing column DNase digestion, while DNA was eluted in 50 µl of elution buffer provided in the kit. DNA/RNA concentrations were measured using a SimpliNano spectrophotometer (GE Healthcare Bio-Sciences Corp., Piscataway, NJ, USA) and stored in a −80°C deep freezer for future use.

### Isolation DNA/RNA from frozen tissues.

For DNA/RNA isolation from tissues, the AllPrep DNA/RNA minikit (Qiagen, Germantown, MD, USA; cat. no. 80204) was used. Approximately 30 mg of frozen tissue was placed into a 2-ml flat-bottom centrifuge tube on dry ice with a 5-mm stainless steel bead (Qiagen; cat. no. 69989) at the bottom. Then, 600 µl buffer RLT plus with 2-mercaptoethanol (βME) at 10 µl βME/ml of RLT plus was added before placing the tube into a TissueLyser LT (Qiagen, USA; cat. no. 69980) set at 50 oscillations/s for 5 min. The lysate was checked for homogeneity and transferred to a QIAshredder (Qiagen; cat. no. 79656). DNA/RNA was then isolated per the kit’s instructions. RNA was eluted in 30 µl of RNase-free water after performing column DNase digestion, while DNA was eluted in 50 µl of buffer EB. DNA/RNA concentrations were measured using a SimpliNano spectrophotometer (GE Healthcare Bio-Sciences Corp., Piscataway, NJ, USA) and stored in a −80°C deep freezer for future use.

### SIV cell-associated RNA/DNA quantification.

Total cell-associated SIV RNA was quantified using RT-ddPCR using 100 ng of RNA, a 1-Step RT-ddPCR Advanced kit for probes (Bio-Rad; cat. no. 1864022), and the same set of primers and probe used for SIV plasma viral load quantification. ddPCR was carried out on a Bio-Rad QX200 AutoDG digital droplet PCR system. In brief, 22 μl of reaction mix was used for droplet generation using a QX200 droplet generator, the ddPCR plate having the emulsified samples was heat sealed with foil (Bio-Rad; cat. no. 181-4040), and amplification occurred in a C1000 Touch thermal cycler (Bio-Rad, CA, USA). After thermal cycling, ddPCR plates were transferred to the QX200 droplet reader (Bio-Rad) for droplet count and fluorescence measurement. Positive droplets with amplified products were separated from negative droplets without target amplicon by applying a fluorescence amplitude threshold, and the absolute quantity of RNA per sample (copies/µl) was determined using QuantaSoft software. For quantification of cell-associated SIV DNA, the same methodology was used as described above without using the reverse transcription step and with 2× ddPCR Supermix for probes (no dUTP) (Bio-Rad; cat. no. 1863024) instead of the 1-Step RT-ddPCR Advanced kit.

### Tat/rev induced limiting dilution assay.

The Tat/rev induced limiting dilution assay (TILDA) was performed as described earlier (85, 86). In brief, enriched CD4^+^ T cells from frozen PBMCs or LN total cells were suspended in complete RPMI at a 2-million cells/ml concentration and then rested at 37°C and 5% CO_2_ for a minimum of 2 h. After resting, cells were divided into two groups: one was stimulated with 100 ng/ml phorbol myristate acetate (PMA) and 1 μg/ml ionomycin, and the other group was left untreated as the control. After 16 h, the cells were counted and then centrifuged at 2,000 rpm for 10 min to stop the reaction. Cells were then serially diluted in complete RPMI medium to the following concentrations: 18,000, 9,000, 3,000, and 1,000 cells/μl. A 96-well PCR plate was divided column-wise in half for the two treatments and then into quarters for the four-dilution sets in 8 replicates. In each well of the plate, 5 µl of master mix was added, and then 1 µl of the appropriate dilution for the corresponding treatment was added to each well. The first PCR mix contained 0.2 µl of Super Script III RT/Platinum *Taq* mix (Invitrogen; cat. no. 2010520), 0.1 µl of Superase RNase inhibitor (Invitrogen; cat. no. 00749138), 2.25 µl of nuclease-free H_2_O (Ambion; cat. no. AM9937), 2.2 µl of tris-EDTA (TE) buffer, 0.125 µl of 10 µM SIVF (5′-CACGAAAGAGAAGAAGAACTCCG-3′; IDT), and 0.125 μl 10 µM SIVR (5′-TCTTTGCCTTCTCTGGTTGG-3′; IDT). To each well of the plate, 5 µl of reaction mix was added. Preamplification settings included reverse transcription for 15 min at 50°C followed by denaturation for 2 min at 95°C and then 24 cycles of amplification at 95°C for 14 s and 60°C for 4 min. Preamplification was conducted in a LifePro thermal cycler (Bioer Technology). After preamplification, 9 µl of qPCR mix containing 5 μl LightCycler 480 Probes Master (Roche; cat. no. 04707494001), 3.4 μl of nuclease-free H_2_O (Ambion; AM9937), 0.2 µl of 20 µM nested FW primer (5′-AGGCTAAGGCTAATACATCTTCTG-3′; IDT), 0.2 µl of 20 µM SIVR (5′-TCTTTGCCTTCTCTGGTTGG-3′; IDT), and 0.2 µl of 5 µM probe (5′-/56-FAM/AAACCCATA/ZEN/TCCAACAGGACCCGG/3IABkFQ/-3′) was added to each well of a new 96-well PCR plate. Then, 1 µl of PCR product from preamplification was added to the corresponding well in the new plate. Real-time PCR was performed in QuantStudio 3 (Applied Biosystems, Waltham, MA, USA) with the following settings: preincubation at 95°C for 10 min followed by 45 cycles at 95°C for 10 s, 60°C for 30 s, and 72°C for 1 s, and then finally a cooling period at 40°C for 30 s.

### Intact proviral DNA assay.

The intact proviral DNA assay was performed as described by Bender et al. (47), including thermal cycling conditions, primers, and probes used in this assay. In brief, 250 to 1,000 ng of DNA was mixed with 10 µl of 2× ddPCR Supermix for probes (no dUTP) (Bio-Rad; cat. no. 863024), 600 nM primers, and 200 nM probes. ddPCR was carried out on a Bio-Rad QX200 AutoDG digital droplet PCR system as described above. To quantify the input cell numbers, a parallel ddPCR reaction for the rhesus macaque RPP30 gene was carried out.

### Macrophage quantitative viral outgrowth assay.

In this study, a modified version of the macrophage (MØ) QVOA was performed on CD11b^+^ macrophages isolated from brain, lung, and liver as described by Avalos et al. (52). In brief, cells were purified using a CD11b^+^ isolation kit per the manufacturer’s recommended protocol (Miltenyi Biotec, Auburn, CA; cat. no. 130-091-100) as described above. The presence of the contaminating CD3^+^ T cells within the enriched CD11b^+^ cells was estimated by quantification of TCRβ as described by Avalos et al. (52). Prior to setting the culture, plates were coated with poly-l-lysine solution (Sigma; NC9778696) for 30 min and then washed twice with PBS. Purified cells were plated in triplicates and in a 10-fold serial dilution in the presence of 10 µM zidovudine (Sigma), 25 nM darunavir (DRV; Janssen), and 5 nM ritonavir (RTV; Merck). BrMφ medium (Dulbecco's modified Eagle medium [DMEM] [Gibco; cat. no. 12491-015], 5% heat-inactivated FBS [Gibco; cat. no. 10437-028], 5% IS giant cell tumor conditioned medium [Irvine Scientific; cat. no. 91006], 100 U/ml penicillin and 100 μg/ml strep [Gibco; cat. no. 15140-122], 70 µg/ml gentamicin [Sigma-Aldrich; cat. no. G1397-10ML], 2 mM l-glutamine [Gibco; cat. no. 35050079], 3 mM sodium pyruvate [Gibco; cat. no. 11360070], and 10 mM HEPES buffer [Gibco; cat. no. 35050079]) was used for brain cells, and complete RPMI medium was used for lung and liver. The culture was then placed in a 37°C 5% CO_2_ incubator for 72 h to permit proper cell adherence. After the 3-day incubation, the antiretroviral-containing medium was removed, and cells were washed with PBS (1×) and then replenished with BrMφ/complete RPMI medium containing 10 ng/ml tumor necrosis factor alpha (TNF-α) (ProSpec; cat. no. CYT-114), 1 µg/ml Pam3CSK4 (Sigma-Aldrich; cat. no. 506350), and 1 µg/ml prostaglandin (Sigma-Aldrich; cat. no. 538904). Additionally, approximately 10^5^ CEMX-174 feeder cells were added to each well. Supernatants were collected on days 5, 7, 10, and 14 post-culture activation and replenished with medium containing activating agents TNF-α, Pam3CSK4, and prostaglandin. RNA was extracted from the supernatants using the QIAamp Viral RNA minikit (Qiagen; cat. no. 52906). Prior to extraction, the supernatants were centrifuged at 2,000 rpm for 5 min, and then 140 µl was aliquoted and used per the kit’s instructions to isolate RNA. The RNA was eluted in 50 µl of AVE buffer. Viral RNA was quantified in culture supernatant using RT-ddPCR as described above. The frequency of replication-competent latent reservoir cells was estimated using the IUPMStats v1.0 infection frequency calculator (87).

### Flow cytometry evaluation of changes in T cell polarization in peripheral blood and lymph nodes.

The details of all fluorochrome-conjugated monoclonal antibodies used in the flow cytometry experiments are listed in Table 3. The single cell suspensions of PBMC and lymph nodes were prepared from samples collected from cART-treated rhesus macaques (*n* = 11) during necropsy. Two million to four million PBMCs were stimulated with PMA and ionomycin at final concentrations of 50 ng/ml and 500 ng/ml, respectively, in complete RPMI 1640 medium (composition described above). An unstimulated condition was included as an experimental control. After 2 h of incubation at 37°C in a humidified 5% CO_2_ incubator, 1 μl of GolgiPlug (BD Biosciences, USA; cat. no. 555029) containing brefeldin A was added to each well together with 1× of monensin (BioLegend, San Diego, CA; cat. no. 420701) and incubated for an additional 4 h. After that, a 1:1,000 dilution of zombie aqua amine reactive dye (BioLegend; cat. no. 423101) was added to the cell suspensions and incubated for 30 min in the dark. The cells were then washed and Fc blockade was carried out using polyclonal anti-human Fc receptor binding inhibitor (eBioscience, USA; cat. no. 16-9161-71). Surface staining was performed using anti-CD45, anti-CD8, anti-CD4, and anti-NKG2A antibodies for panel 1 and using anti-CD25 and anti-CD127 antibodies for panel 2 (Treg panel); cells were incubated for 1 h at room temperature in the dark. Thereafter, for panel 1, cells were fixed using a 2% paraformaldehyde (PFA) solution for 30 min, and permeabilization was performed using a 1× BD perm/wash solution (BD Biosciences; cat. no. 554723) for 15 min at 4°C. Then, anti-CD3, anti-IFN-γ, anti-TNF-α, and anti-IL-17 antibodies were added, and cells were incubated at 4°C for 15 min, washed, and resuspended in PBS. For the Treg panel, fixation and permeabilization were performed using a 1× solution of FoxP3/transcription factor Fix/Perm concentrate (4×) (Tonbo Biosciences, San Diego, CA; cat. no. TNB-1020-L050) that was diluted using the 1× FoxP3/transcription factor Fix/Perm diluent (Tonbo Biosciences; cat. no. TNB-1022-L160). Following this, anti-CD3, anti-IL-4, and anti-FoxP3 antibodies were added, and cells were incubated at 4°C for 15 min, washed, and resuspended in PBS.

**TABLE 3:** Details of all fluorochrome-conjugated monoclonal antibodies used in the flow cytometry experiments

Antibody	Fluorophore^*a*^	Clone	Vendor	Catalog no.
CD45	BV786	D058-1283	BD Horizon	563861
CD3	AF700	SP34-2	BD Pharmingen	561805
CD3	APC-Cy7	SP34-2	BD Pharmingen	557757
CD4	PE-CF594	L200	BD Biosciences	562402
CD4	AF700	L200	BD Pharmingen	560836
CD8	BV510	SK1	BD Horizon	563919
CD20	BUV805	2H7	BD Horizon	612905
CD25	BV421	BC96	BiolLegend	302612
NKG2a (CD159a)	PC7	Z1999	Beckman coulter	B10246
CD127	PC5	R34.34	Beckman coulter	A64617
CD38	APC	OKT10	NHP Reagent resource	NA^*b*^
CD95	FITC	DX2	BD Pharmingen	556640
HLA-DR	PE-Texas Red	TU36	Life Technologies	MHLDR17
IFN-γ	PE	B27	BD Pharmingen	562016
IL-4	FITC	MP4-25D2	BD Biosciences	554484
IL-17	APC	eBio64Dec17	Invitrogen	17-7179-42
TNF-α	FITC	Mab11	BD Pharmingen	554512
FOXP3	APC	PCH101	eBioscience	17-4776-42
CXCR5	PE	710D82.1	NHP Reagent resource	NA
CXCR5	BV421	J25D4	Biolegend	356919
PD-1	PerCP/Cy5.5	EH12.2H7	Biolegend	329913
CTLA-4	PE-Cy5.5	BNI3	Biolegend	369607
CXCR3	BUV 393	1C6	BD Biosciences	565223
CCR4	PE	1G1	BD Biosciences	561110
CCR6	PE-Cy7	11A9	BD Pharmingen	560620

*^a^* APC, allophycocyanin; PE, phycoerythrin; FITC, fluorescein isothiocyanate.

b NA, not available.

For LN cell suspensions, 2 million to 4 million cells were taken, zombie UV viability dye was added, and Fc receptor blockade was performed as described above. Then, in the T cell polarization panel, anti-CXCR5, anti-CXCR3, anti-CCR4, and anti-CCR6 antibodies were added, and in the Treg panel, anti-CXCR5 antibody was added. After that, cells in both panels were incubated at 37°C for 30 min. Following this, ani-CD45, anti-CD3, anti-CD4, anti-CD8, anti-CD20, anti-CD95, anti-CD38, anti-PD1, and anti-human HLA-DR antibodies were added in the polarization panel, while in the Treg panel, anti-CD25 and anti-PD1 antibodies were added. After that, cells in both panels were incubated at room temperature for 30 min, washed, and fixed. For the Treg panel, intracellular staining was performed as described above for PBMCs using anti-FoxP3 and anti-CTLA4 antibodies. All events were acquired using the BD Fortessa X450, and data were analyzed using FlowJo version 10.6. Fluorescence-minus-one (FMO) controls were included as cutoffs for placement of gates when handling nondiscriminatory variables.

### Statistical analysis.

Graph Pad Prism 8.0 software was used to carryout statistical comparisons and plot the figures. Descriptive statistics were presented to summarize continuous variables. Wilcoxon rank sum tests were used to determine the statistically significant difference in continuous outcomes between morphine and saline groups for flow cytometry data and other experimental data. All *P* values of less than 0.05 are considered statistically significant.
